# Ionic-Liquid-Engineered Chitin Nanofiber Supports for Low-Loading Pd Catalysts

**DOI:** 10.3390/polym18151872

**Published:** 2026-07-30

**Authors:** Oleksandra Zavgorodnya, Hemant Choudhary, Rajkumar Kore, Julia L. Shamshina, Robin D. Rogers

**Affiliations:** 1 Department of Chemistry and Biochemistry, The University of Alabama, Tuscaloosa, AL 35487, USA; 2 Department of Bioresource and Environmental Security, Sandia National Laboratories, Livermore, CA 94551, USA; 3 Fiber and Biopolymer Research Institute (FBRI), Texas Tech University, Lubbock, TX 79409, USA; 4 Department of Chemistry and Biochemistry, Texas Tech University, Lubbock, TX 79409, USA; 5 525 Solutions, Inc., P.O. Box 2206, Tuscaloosa, AL 35403, USA

**Keywords:** ionic liquid (IL), chitin, electrospinning, nanofiber supports, Pd catalyst, Suzuki–Miyaura coupling

## Abstract

This study reports an ionic-liquid (IL)-enabled strategy for engineering chitin nanofiber supports that allows for the formation of monometallic Pd, Ag, and Au catalysts, as well as Pd–Au co-loaded catalyst. High-molecular-weight chitin, extracted from shrimp shells using 1-ethyl-3-methylimidazolium acetate ([C_2_C_1_im][OAc]), is regenerated and electrospun into free-standing nanofiber mats, followed by partial deacetylation of the accessible nanofiber interface to introduce primary amine functionalities while largely preserving the chitin crystalline framework. Metal precursors were first immobilized at the modified chitin nanomat interface at pH ≈ 2, followed by nanoparticle formation upon exposure to alkaline borate buffer (pH ≈ 9.2–10). STEM analysis shows particle sizes of Pd 3.2 ± 1.0 nm, Au 6.4 ± 2.6 nm, and Ag 2.6 ± 1.2 nm. The co-loaded Pd–Au sample likewise exhibited nanoscale, well-dispersed particles with no large aggregates observed within the examined STEM regions. Catalytic performance is evaluated using the Suzuki–Miyaura coupling of bromobenzene with phenylboronic acid as a benchmark reaction. The Pd–chitin nanomats achieve >99% conversion and ≥99% selectivity at gravimetrically estimated Pd loadings as low as 0.012–0.017 mol% under the conditions examined.

## 1. Introduction

Metal nanoparticles (NPs) supported on polymeric matrices have attracted significant attention due to their diverse applications in fine-chemical synthesis, energy conversion, and environmental remediation [1,2,3,4,5,6]. Their unique physicochemical properties, such as high surface-to-volume ratio, tunable electronic structure, and ability to stabilize transition-metal active sites, enable superior catalytic performance compared with bulk metals [7]. Among these systems, polymer-supported metal catalysts are especially appealing because they offer catalyst recovery, recyclability, and product selectivity, all essential for industrial implementation [1,8,9]. Bimetallic NPs often outperform monometallic ones owing to synergistic electronic and geometric effects that modulate adsorption energies and activation barriers [10].

Traditionally, supported metal catalysts have been fabricated using silica, poly(amidoamine) (PAMAM) dendrimers, hyperbranched polymers, carbon nanofibers, metal–organic frameworks (MOFs), and carbon nanotubes (CNTs) as matrices [11,12,13,14,15,16,17,18], using conventional methods such as deposition–precipitation, co-precipitation, or high-temperature thermal reduction under H_2_ or N_2_ [19]. However, these approaches often result in NP sintering, broad size distributions, and poorly controlled surface compositions, particularly on rigid inorganic supports [20,21]. In contrast, in situ formation of metal NPs within polymeric matrices allows finer control over NP size and dispersion, as the polymer’s functional groups act simultaneously as stabilizing and reducing agents. Still, many reported in situ methods rely on strong reducing agents, generating hazardous by-products and limiting scalability [22,23,24].

As catalysis increasingly aligns with green chemistry principles, there is growing demand for renewable, biodegradable, and environmentally benign supports that maintain catalytic efficiency while minimizing persistent waste [25]. Naturally derived biopolymers, such as cellulose, alginate, and chitosan offer attractive alternatives to synthetic matrices because they provide abundant functional groups capable of coordinating and stabilizing metal ions [26,27,28,29,30,31,32,33,34,35].

Cellulose-supported NPs have already shown high activity in key cross-coupling reactions under mild and recyclable conditions [36,37], and their surface chemistry is dominated by hydroxyl groups (–OH), which stabilize metal species through hydrogen bonding, electrostatic interactions (when charged groups are present), and coordination via oxygen donors [38]. Cellulose support is inherently optimized for O-donor and hydrogen-bonding interactions and has demonstrated strong performance in coupling reactions [38,39]. However, for noble-metal catalysts (e.g., Pd, Au, Pt), catalytic performance is also governed by how effectively the support (i) captures ionic metal precursors from solution, (ii) controls nucleation and growth into small nanoparticles, and (iii) suppresses post-synthesis deactivation pathways such as aggregation, sintering, and metal leaching.

In this context, supports incorporating nitrogen-donor sites, particularly surface-accessible primary amines, often exhibit stronger metal binding and improved nanoparticle anchoring relative to hydroxyl-dominated supports, enabling chelation geometries and binding strengths not accessible on hydroxyl-only surfaces [40,41,42]. This stronger coordination promotes improved dispersion and stabilizes noble-metal nanoparticles against migration-induced coalescence under mild synthesis and reaction conditions [40,41,43].

Consistent with the stronger metal–support interactions enabled by nitrogen-donor coordination, chitosan, produced by deacetylation of chitin, has been widely explored as a biopolymeric support due to its low cost, biodegradability, and strong chelating ability [44,45,46,47]. However, extensive bulk deacetylation converts chitin into a more amorphous and mechanically weaker material, limiting its suitability as a robust catalytic support. Native chitin, in contrast, contains nitrogen in acetamide groups that are less nucleophilic and exhibit weaker metal coordination but provides superior mechanical strength and dimensional stability due to its crystalline structure.

Nanofibrous architectures can provide highly accessible interfaces and open porous networks advantageous for catalytic applications [48,49,50]. The only example of a nanofibrillated chitosan support for Pd-catalyzed cross-coupling appears to be a glutaraldehyde-crosslinked electrospun chitosan nanofiber mat loaded by adsorption of Pd(II) salts, which enabled Heck coupling under polar aprotic conditions and demonstrated the promise of recoverable fibrous biopolymer architectures for heterogeneous catalysis [51]. However, this precedent also demonstrates key limitations that remain unresolved for next-generation green catalytic materials: chitosan’s bulk deacetylation can compromise native polysaccharide crystallinity and mechanical properties, necessitating chemical crosslinking; Pd is introduced primarily as coordinated Pd(II) rather than as structurally controlled, surface-anchored Pd^0^ nanodomains; and catalyst efficiency is still achieved at comparatively higher precious-metal loadings than would be ideal for low-loading catalyst design [51]. These considerations motivate an alternative design principle in which nitrogen-donor functionality is introduced preferentially at the accessible nanofiber interface, maximizing metal binding and nucleation control, while the underlying polysaccharide core retains the structural integrity needed for robust handling and recovery.

A major limitation of chitin is its insolubility in water and most organic solvents, which restricts conventional processing; however, this same property can be advantageous by enabling its use as a heterogeneous support. Ionic liquids (ILs, salts liquid below 100 °C) have enabled direct dissolution and molecular-level regeneration of high-molecular-weight chitin [52] by disrupting its hydrogen-bonding network while preserving polymer integrity. This allows fabrication of diverse morphologies, including fibers, films, and beads [53,54,55,56,57,58,59,60,61,62]. Our group achieved scalable electrospinning of chitin from ILs, yielding nanofiber networks with diameters below 23 nm and uniform distribution across large areas [63].

Because only the accessible fiber interface requires free amino groups for metal coordination, we hypothesized, building on our earlier work [64], that partial deacetylation of the chitin nanofiber surface would provide an advantageous support architecture. This design enriches the accessible fiber interface with amino functionality while largely preserving the crystalline chitin framework, thereby combining (i) interfacial coordination sites for effective nanoparticle anchoring with (ii) retained bulk crystallinity and mechanical integrity. Such architecture is particularly attractive for recoverable heterogeneous catalysis [40,41]. Upon mild reduction, metal ions are expected to yield uniformly dispersed metal nanoparticles anchored to the surface, thereby maximizing accessible catalytic sites and preventing aggregation. The largely preserved chitin-rich core contributes mechanical durability during catalytic operation, producing a fiber that is chemically active at its surface yet inert and stable within, effectively merging the advantages of homogeneous catalysis (high activity) and heterogeneous catalysis (ease of recovery) within a single renewable nanoscale system.

We therefore sought to develop a green, mild route for synthesizing monometallic and co-loaded nanoparticles within IL-processed, partially deacetylated chitin nanofiber mats. Boric acid and borate-based media have been recurrently employed in the controlled synthesis of Pd^0^ nanoparticles on amine-bearing supports [65,66,67], where they regulate pH. Across a range of systems, these conditions yield ultrasmall (typically 3–6 nm), uniformly dispersed M^0^ particles through slow reduction of M^n+^ precursors. Herein we report an integrated approach combining IL-based chitin processing with mild, aqueous in situ nanoparticle synthesis, offering a renewable and recyclable platform for heterogeneous catalysis.

## 2. Experimental

### 2.1. Materials

Dried shrimp shells were obtained from the Gulf Coast Agricultural and Seafood Cooperative (Bayou La Batre, AL, USA). At the facility, shells were first mechanically dewatered using a screw press to remove most of the moisture and then subjected to thermal drying in a fluidized bed dryer at temperatures up to 160 °C until the residual moisture content was below 5 wt%. The dried material was subsequently ground with an IKA Works Universal Grinding Mill (Wilmington, NC, USA) and sieved to obtain particles of <250 µm. The resulting shrimp shell powder was further oven-dried at 80 °C for 24 h using a Precision Scientific Econotherm Laboratory Oven, Model 1025 (Winchester, VA, USA).

Sodium tetrachloropalladate(II) (Na_2_PdCl_4_, ~36% Pd) and potassium tetrachloroaurate(III) (KAuCl_4_, 98%) were purchased from Fisher Scientific (Waltham, MA, USA). Silver nitrate (AgNO_3_, ≥99%), bromobenzene (99%), and phenylboronic acid (95%) were obtained from Sigma-Aldrich (St. Louis, MO, USA) and used as received. Borate buffer solution (pH 10, Acros Organics (Geel, Belgium)) and anhydrous potassium carbonate (K_2_CO_3_, 99%) were supplied by Fisher Scientific (Waltham, MA, USA). Ethanol (95%) and dichloromethane (≥99.5%) were obtained from Sigma-Aldrich (St. Louis, MO, USA) and were used without further purification. Deionized (DI) water (resistivity 16.82 MΩ·cm at 25 °C) was obtained from a Culligan deionizer (Northbrook, IL, USA). The ionic liquid (IL) 1-ethyl-3-methylimidazolium acetate ([C_2_C_1_im][OAc], purity > 95%) was purchased from IoLiTec, Inc. (Tuscaloosa, AL, USA).

### 2.2. Preparation of Chitin Support

*Chitin Extraction from Biomass.* Shrimp shells (2 wt%) were dissolved in [C_2_C_1_im][OAc] using microwave-assisted heating (2 s pulses with manual stirring for 6 min; 10 s pulses during the first 30 s). The resulting viscous solution was centrifuged at 3000 rpm for 20 min to remove undissolved residues. The clarified supernatant was collected for regeneration of chitin.

*Regenerated Chitin.* The decanted shrimp shell solution (60 g) was coagulated in 1 L of DI water under continuous stirring and left overnight to remove the IL. The coagulated chitin was isolated by centrifugation, washed repeatedly (10 cycles) with fresh DI water using sonication and centrifugation (3000 rpm, 15 min), and oven-dried at 60 °C. The dried chitin was ground and sieved to obtain a powder fraction with particle size <125 µm.

The MW of chitin (3.9 ± 1.4 × 10^6^ Da) was determined using static light scattering (SLS) in solution of the same IL, as shown in [68].

*Chitin Re-dissolution.* The dried, size-selected regenerated chitin powder was re-dissolved in [C_2_C_1_im][OAc] at 90 °C under continuous stirring to prepare dilute solutions suitable for electrospinning. Chitin and ionic liquid were combined to obtain a 0.3 wt% chitin/[C_2_C_1_im][OAc] suspension, which was heated at 90 °C until a clear, homogeneous dope was formed (typically 24 h). This thermally prepared 0.3 wt% solution was directly used for electrospinning.

*Electrospinning of Chitin.* Regenerated chitin solutions in [C_2_C_1_im][OAc] (0.3 wt%) were electrospun using a custom-built multi-needle system [63]. Approximately 50 g of chitin–IL solution was loaded into a feed reservoir connected to the spinneret and subjected to an applied voltage of 25–26 kV (UltraVolt, Ronkonkoma, NY, USA). The solution was electrospun directly into a DI water coagulation bath positioned 9.5 cm below the spinneret. The as-spun mats were thoroughly washed in DI water to remove residual IL and air-dried on Teflon-coated stainless-steel mesh (100 mesh, TVP Inc., Berkeley, CA, USA).

*Deacetylation of Electrospun Chitin Mats.* The electrospun mats were immersed in 1.25 M NaOH at 80 °C for 8 h to partially deacetylate the chitin surface and introduce primary amine groups [64]. After 8 h, the fibers appeared to lighten in color, and the liquids were decanted. Each set of DA mats was washed with DI water a total of three times (3 × 100 mL) with swirling until neutral pH (~7). No visible changes to the structure of the fibers were observed during this process. The mats were then rinsed with weak acid (0.1 M HCl, pH~2) to protonate the amino groups and prepare the surface for metal ion complexation.

*Synthesis of Metal Nanoparticles (NPs).* Deacetylated chitin mats were immersed in aqueous solutions of sodium tetrachloropalladate(II) (1 mM, pH 2), silver(I) nitrate (1 mM, pH 2), or sodium tetrachloropalladate(II)/potassium tetrachloroaurate(III) mixture (0.5 mM/0.5 mM, pH 2) for 72 h in the dark to ensure complete surface saturation. After loading, the mats were washed with pH 2 water (adjusted with 0.1 M HCl) to remove unbound ions. Reduction of metal ions was carried out by immersing the loaded mats in borate buffer (15 mL, pH 10) for 11 days in the dark. The mats were subsequently washed with pH 2 water (adjusted with 0.1 M HCl) and finally with DI water. The resulting NP–chitin mats were stored in 95% ethanol until further use.

### 2.3. Methods

*Powder X-ray Diffraction (pXRD).* pXRD analysis of extracted chitin was conducted using a Rigaku HD 2711N diffractometer (Rigaku, Tokyo, Japan) equipped with a Ni-filtered Cu Kα radiation source (λ = 1.542 Å). Measurements were collected at room temperature under an operating voltage of 40 kV and a current of 44 mA. Diffraction patterns were recorded over a 2θ range of 5–50° at a scan rate of 1° min^−1^. Raw data were processed using OriginPro, version 9.1 (OriginLab Corporation, Northampton, MA, USA) for background subtraction, smoothing, and baseline correction. Peak identification, indexing, and further crystallographic analysis were performed using the same software, Peak Analyzer module, allowing comparison of experimental reflections with standard α-chitin crystallographic patterns.

*Fourier Transform Infrared Spectroscopy (FTIR).* FTIR was performed using an ATR-FTIR Bruker Alpha II spectrometer (Bruker Optics, Billerica, MA, USA). Samples were placed directly onto a chemically inert diamond crystal stage and gently pressed using the pressure clamp to ensure optimal contact and reproducibility. Spectral data were collected over the wavenumber range of 400–4000 cm^−1^, with 64 co-added scans acquired at a resolution of 4 cm^−1^. All spectra were recorded and processed using OPUS, version 7.0 (Bruker Optics GmbH, Ettlingen, Germany), including baseline correction and normalization, enabling reliable identification of the functional groups characteristic of chitin.

*Solid-State ^13^C multi-Cross-Polarization Magic Angle Spinning Nuclear Magnetic Resonance (multiCP/MAS NMR) Spectroscopy.* NMR spectra were acquired on a 400 MHz Varian VNMRS spectrometer using a 4 mm double-resonance Varian Chemagnetics T3 probe (Agilent Technologies, Santa Clara, CA, USA). Approximately 30 mg of sample was center-packed into zirconia rotors and spun at 8 kHz. Quantitative ^13^C spectra were obtained using the multiple-cross-polarization (multiCP) methodology previously established and validated for chitin purity analysis [69].

Standard single-contact CP/MAS spectra were also acquired for comparison using a recycle delay of 3 s, a contact time of 1000 μs, and a ^1^H spin-lock field of approximately 60 kHz. Additional CP spectra were acquired with a contact time of 8000 μs to permit direct comparison with the multiCP spectra, for which the cumulative CP contact time was 8000 μs. A total of 2872 scans were acquired for each sample, corresponding to an acquisition time of approximately 2 h 24 min. SPINAL-64 ^1^H decoupling with an rf field of 90 kHz was applied during acquisition. CP build-up curves were additionally obtained using contact times ranging from 30 μs to 8 ms, with 512 scans acquired at each contact time.

*Purity Determination by SS ^13^C multiCP/MAS NMR.* Sample purity was determined from the quantitative SS ^13^C multiCP/MAS NMR spectra, using the previously validated integration method for chitin/protein mixtures [69], according to the following Equation:

$$\%\mathrm{Purity}=100\times \frac{\sum I_{\mathrm{target}\;\mathrm{compound}\;(\mathrm{chitin})}}{\sum I_{\mathrm{total}}}$$

where $I_{\mathrm{target}\;\mathrm{compound}}$ represents the integrated area of resonances corresponding to characteristic chitin carbon signals, and $I_{\mathrm{total}}$ represents the integrated area of all observed resonances, including contributions from chitin and residual impurities.

*Molecular Weight Determination by SLS.* Weight-average molecular weight (M_w_) was determined by static light scattering (SLS). A series of chitin solutions with concentrations ranging from 0.9 to 9.0 mg g^−1^ were prepared by direct thermal dissolution of dried, ground chitin in 1-ethyl-3-methylimidazolium acetate ([C_1_C_2_im][OAc]) at 90 °C for 24 h under constant magnetic stirring. The refractive indices of the chitin solutions were measured at 25 °C using an Abbemat 500 refractometer (Anton Paar, Ashland, VA, USA) after equilibration for 1 h. Plots of refractive index versus concentration were used to determine dn/dc values, which were averaged and subsequently used to calculate the optical constant (K) for SLS analysis.

For light scattering measurements, approximately 1 mL of each solution was transferred into 3.5 mL rectangular optical glass cuvettes (10 mm path length). Static scattering intensities of the chitin solutions, neat ionic liquid (background), and a toluene standard were measured using a Zetasizer Nano S instrument (Malvern Instruments, Malvern, UK) equipped with a He–Ne laser (λ_0_ = 633 nm) at a scattering angle of 173° and a controlled temperature of 25 °C. For each sample, ten consecutive measurements (10 s each) were collected with a 10 s equilibration period between runs. Using the instrument software, background-corrected Rayleigh ratios (R_θ_) were calculated. These values were used to construct Debye plots of K_c_/R_θ_ versus concentration, and the intercept at c → 0 was used to determine M_w_. 

*Mercury intrusion porosimetry*. Mercury intrusion porosimetry was performed on representative electrospun chitin mats using a Quantachrome PoreMaster instrument (Boynton Beach, FL, USA). Measurements were conducted using a mercury surface tension of 480 erg cm^−2^ and a mercury contact angle of 140°. Mercury intrusion volume was normalized to sample mass. Porosity was calculated using a helium density of 1.44 g cm^−3^. The analyzed sample masses were 0.0185 g (trial 1) and 0.0157 g (trial 2).

*Ultraviolet–Visible (UV–Vis) Spectroscopy*. Spectra were recorded on a PerkinElmer Lambda XLS spectrophotometer (Shelton, CT, USA) over the range of 200–800 nm. Air-dried NP–chitin mats were mounted on quartz slides (0.8 × 2.2 cm) and placed in 1 cm quartz cuvettes. Spectra of precursor salt solutions were obtained under identical conditions for comparison.

*Scanning Transmission Electron Microscopy–Energy-Dispersive X-ray Spectroscopy (STEM-EDX).* Nanoparticle morphology and size distributions were determined using a Tecnai 20 TEM (FEI, Hillsboro, OR, USA) operated at 200 kV and equipped with an EDX detector. The NP–chitin mats were oven-dried, ground, and deposited from ethanol onto carbon-coated copper grids (Ultrathin C Film on Holey Carbon Support Film, 400 mesh, Ted Pella, Inc. (Redding, CA, USA)). Samples were air-dried before imaging.

FFT analysis of individually cropped HRTEM nanoparticle images was carried out in FIJI (ImageJ, version 1.49) using the instrument-calibrated pixel size of 0.03805 nm to determine lattice spacings and crystallographic orientations. The measured lattice spacing of approximately 0.23 nm lies between the reported fcc Au(111) (~0.235 nm) and Pd(111) (~0.224 nm) spacings.

*Gravimetric determination of Pd loading.* Prior to metal loading, the wet chitin nanomats were divided into three approximately equal portions. One portion was dried at 100 °C to constant mass and weighed to determine the dry-mass equivalent of an individual nanomat portion. A second portion was maintained in the wet state and subjected to Pd precursor loading, reduction, and extensive aqueous washing to remove unbound species. Following washing, the Pd-loaded mat was dried at 100 °C to constant mass and weighed. The increase in dry mass relative to the dry-mass equivalent determined from the untreated portion was attributed to retained Pd and used to estimate the Pd loading, which was 2 wt%. This value was subsequently used to estimate the Pd mass and mol% Pd employed in the catalytic experiments. The third portion was used for catalytic experiments.

*Gas Chromatography–Mass Spectrometry (GC–MS).* Catalytic reaction products were analyzed using a Waters Autospec NT mass spectrometer coupled with an HP6890 GC inlet (Waters, Milford, MA, USA). The temperature program began at 50 °C (1 min hold), ramped at 10 °C·min^−1^ to 200 °C, and was held for 10 min. A Zebron ZB-5MS capillary column (30 m × 0.25 mm i.d., 0.25 µm film thickness; Phenomenex, Inc., Torrance, CA, USA) was used, with the EI source temperature set to 220 °C. Samples were prepared in dichloromethane.

*Catalytic Tests in Suzuki Cross-Coupling.* Reactions were performed in 3 mL of ethanol/water (2:1 *v*/*v*) containing 0.50 mmol bromobenzene, 0.75 mmol phenylboronic acid, Pd–chitin catalyst, and 1.00 mmol K_2_CO_3_. The Pd–chitin catalyst mass was varied to provide estimated Pd quantities of 1.0, 2.3, 6.4, and 9.2 µg per reaction, corresponding to calculated Pd loadings of 0.0019, 0.0043, 0.0120, and 0.0173 mol%, respectively, based on the gravimetrically estimated Pd content of the nanomats. The reaction mixture was maintained at 70 °C for 12 h under identical conditions (without stirring). After reaction completion, the mixture was extracted with 3 mL dichloromethane to separate the organic phase. An aliquot of the bottom layer was diluted with 4–5 mL dichloromethane and analyzed by GC–MS. Conversions and selectivities were calculated from peak area integration.

## 3. Results and Discussion

### 3.1. Preparation and Structural Characterization of Chitin Nanomats, Surface Deacetylation, and Electrostatic Adsorption Under Acidic Conditions

Chitin was extracted from shrimp shells using the ionic liquid (IL) 1-ethyl-3-methylimidazolium acetate ([C_2_C_1_im][OAc]), following a previously established microwave-assisted extraction method [52]. This ionic liquid was selected because of its established ability to dissolve high-molecular-weight chitin by disrupting the extensive hydrogen-bonding network of the polysaccharide without requiring prior derivatization [53,54,55,56,57,58,59,60,61,62,63,64]. Following dissolution, chitin can be regenerated by aqueous coagulation and subsequently processed into fibers, films, beads, and other morphologies. Of particular relevance to the present work, [C_2_C_1_im][OAc]-chitin solutions have previously been demonstrated to be suitable for electrospinning high-molecular-weight chitin into nanofibrous networks [63]. Thus, use of this ionic liquid provides an integrated route from shrimp-shell-derived chitin to the nanofibrous support architecture required for subsequent surface modification and metal immobilization.

In this work, [C_2_C_1_im][OAc] disrupts the extensive hydrogen-bonding network of chitin and solvates the polysaccharide fraction, with the acetate anion playing a major role in hydrogen-bond disruption. Upon regeneration in water, chitin precipitates, while soluble proteinaceous components and residual IL are removed through repeated aqueous washing. Insoluble inorganic mineral residues are separated from the chitin-containing solution by centrifugation and/or filtration prior to regeneration. Thus, the IL treatment enables chitin dissolution and regeneration, while proteins and mineral impurities are removed through the associated washing and solid–liquid separation steps. The regenerated chitin was recovered by water coagulation and thoroughly washed to remove residual IL [68].

The chemical identity of the IL-extracted chitin was confirmed by ^13^C multiple-cross-polarization magic-angle spinning nuclear magnetic resonance (^13^C multiCP/MAS NMR) spectroscopy (ESI, Figure S1). Unlike conventional single-contact CP/MAS experiments, the multiCP approach provides quantitative ^13^C signal intensities through repeated polarization-transfer periods [70,71,72] and has been validated for quantitative determination of chitin content and purity [73]. The method used here was previously calibrated using mass-based chitin/protein mixtures and shown to yield chitin-content values consistent, within experimental error, with those obtained by an independent chemical analysis [69]. On this basis, the extracted material contained >95% chitin.

Powder X-ray diffraction (pXRD) provided complementary evidence for the absence of detectable crystalline mineral residues (Figure S2). The diffractogram exhibited the characteristic reflections at 2θ = 9.22°, 12.84°, 19.44°, and 26.22°, consistent with previously reported diffraction patterns for this type of chitin [68]. Attenuated Total Reflectance Fourier transform infrared (ATR-FTIR) spectroscopy further confirmed the characteristic chemical structure of the extracted chitin (Figure S3) [68]. Characteristic bands included C–H stretching vibrations of CH, CH_2_, and CH_3_ groups at 3089, 2929, and 2877 cm^−1^, respectively; amide I absorptions at 1654 and 1628 cm^−1^; an amide II band at 1547 cm^−1^; an amide III band at 1306 cm^−1^; and an asymmetric N–H stretch at 3263 cm^−1^, all in agreement with literature values [74].

For the fabrication of nanofibrous mats, extracted chitin was thermally re-dissolved at 0.3 wt% in the same IL to obtain a homogeneous, viscous spinning dope suitable for electrospinning [75]. Such low concentration provided a spinning dope exhibiting electrospinning behavior typical for previously reported IL–chitin solutions. Electrospinning was performed using a custom multi-needle linear spinneret with ~50 g of IL–chitin solution loaded into the feed reservoir. The operating parameters were carefully optimized to maintain jet stability and continuous fiber formation, as previously described [76]: applied voltage 25–26 kV, needle-to-collector distance 9.5 cm, and ambient pressure. The fibers were directly collected in a DI water coagulation bath, which simultaneously induced polymer regeneration and removed the IL from the forming filaments.

Following coagulation and extensive washing, ^13^C multiCP/MAS NMR spectra of the regenerated nanofibrous mats exhibited the same characteristic resonances as the extracted chitin (Figure S4), confirming preservation of the chitin chemical structure during IL processing and electrospinning. pXRD analysis of the regenerated mats as expected showed no evidence of contamination (Figure S5) and ATR-FTIR spectra of the regenerated mats (Figure S6) retained the characteristic features of α-chitin, including the amide I (1648/1626 cm^−1^), amide II (1547 cm^−1^), and amide III (1305 cm^−1^) bands. Complete removal of [C_2_C_1_im][OAc] was confirmed by the absence of characteristic imidazolium vibrations, including imidazolium C2–H bending at ~1385 and C=N stretching at 1565 cm^−1^ modes, indicating effective regeneration and solvent removal.

**Figure 1:**
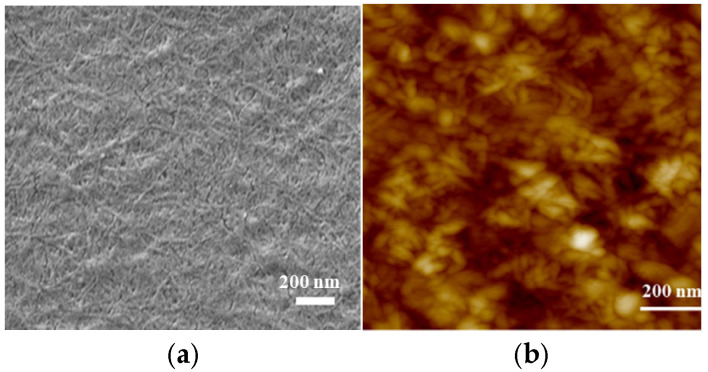
Surface morphology of electrospun chitin nanofibrous mats. (**a**) SEM micrograph showing a highly uniform and interconnected porous network suitable for subsequent metal nanoparticle immobilization. (**b**) AFM topography image revealing continuous fibers with diameters in the range of 20–25 nm.

The obtained chitin nanofibrous mats exhibited a highly uniform, interconnected porous morphology, ideal for subsequent metal nanoparticle immobilization. Scanning Electron Microscopy (SEM) and Atomic Force Microscopy (AFM) analyses (Figure 1) revealed continuous fibers with representative fiber diameters in the range of 20–25 nm in the imaged regions.

To quantitatively characterize the porous structure, mercury intrusion porosimetry data for representative electrospun chitin mats were collected. The mats exhibited total intruded pore volumes of 23.46–24.08 cm^3^ g^−1^, porosities of 97.13–97.20%, and mercury-intrusion-derived surface areas of 5.10–5.35 m^2^ g^−1^. The volume-distribution modes occurred at pore diameters of approximately 17.5–18.6 µm (Table S1 and Figure S7), confirming a highly open, predominantly macroporous architecture.

**Scheme 1:**
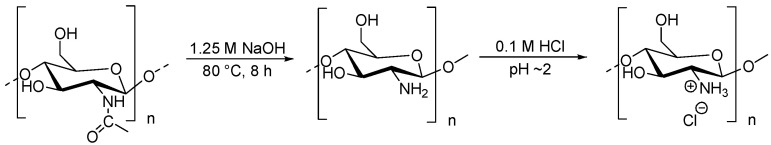
Proposed protonation of accessible amino groups at the partially deacetylated chitin interface prior to electrostatic adsorption of anionic metal precursors.

Electrospun chitin nanomats were partially deacetylated in 1.25 M NaOH at 80 °C for 8 h using a surface-modification protocol previously developed by our group for regenerated chitin fibers [64] (Scheme 1). In that earlier study, the insolubility of chitin in aqueous media was exploited to preferentially modify the accessible fiber surface while retaining a predominantly chitinous interior. Comparison of ATR-IR spectra of intact and ground fibers revealed differences between the accessible surface and bulk material, while surface-sensitive XPS analysis of the outer approximately 1–12 nm showed pronounced changes in the N 1s region consistent with conversion of acetylated nitrogen environments to amine-containing functionality after alkaline treatment [64]. These results established the feasibility of producing a chemically modified, amine-enriched interface while largely preserving the underlying chitin structure.

**Figure 2:**
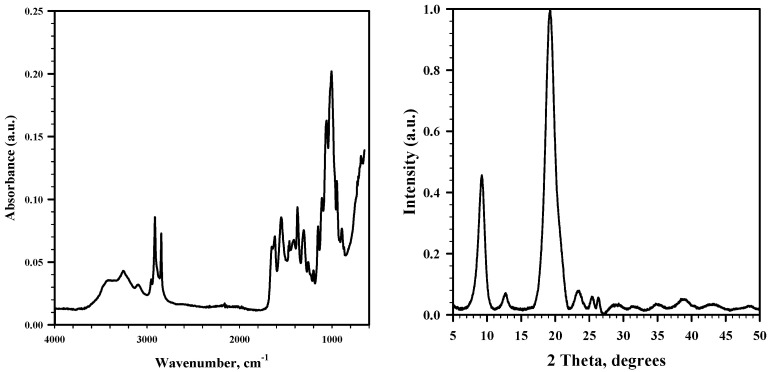
ATR-FTIR (**left**) and pXRD data (**right**) for the deacetylated nanomats.

The same partial deacetylation of the accessible nanofiber interface concept was applied to the electrospun chitin nanomats in the present study. ATR-FTIR and pXRD analyses of the treated nanomats (Figure 2) showed retention of the characteristic chitin vibrational features and crystalline framework, indicating that the alkaline treatment did not measurably disrupt the bulk chitin structure. However, because ATR-FTIR and pXRD are not surface-specific and the absolute degree of surface deacetylation and surface-accessible amine density were not quantified in the present study, these data do not independently establish the spatial distribution or concentration of amino groups across the nanofiber cross-section. Accordingly, we describe the treated material as possessing a partially deacetylated, amine-enriched accessible nanofiber interface while retaining a predominantly chitin-like bulk framework, consistent with the previously established surface-modification methodology [64]. The modification was validated functionally through subsequent metal-binding experiments.

Following partial deacetylation and washing, the modified nanomats were equilibrated with 0.1 M HCl (pH ~2) to protonate accessible amino groups prior to exposure to the metal precursors (Scheme 1). The mats retained their free-standing morphology during this acid treatment and subsequent acidic metal-precursor loading and washing. This qualitative stability is consistent with retention of a predominantly chitin-rich bulk rather than extensive conversion to acid-soluble bulk chitosan; quantitative acid-solubility or mass-loss measurements were not performed.

Under these acidic conditions, accessible amino groups are expected to exist predominantly as –NH_3_^+^ sites. For Pd(II), introduced as the anionic tetrachloropalladate complex [PdCl_4_]^2−^, initial association with the modified chitin interface is therefore proposed to occur primarily through electrostatic attraction and ion pairing with protonated amine sites rather than through direct Pd–N coordination. This proposed interfacial association provides a basis for retention of the Pd(II) precursor at the partially deacetylated nanofiber interface prior to the subsequent alkaline treatment.

### 3.2. Proposed Pathway for In-Situ Formation of Nanoparticles on Chitin Supports

The following mechanistic description is inferred from the established literature on Pd(II) hydrolysis in aqueous media and on the coordination, immobilization, and stabilization of palladium species by amine-containing polysaccharide surfaces [77,78,79,80,81,82,83,84]. It is intended to rationalize the experimentally observed formation of Pd-containing nanoparticles on partially deacetylated chitin mats. Direct observation of transient coordination or redox intermediates was not undertaken in the present study; therefore, the individual steps described below should be regarded as a plausible mechanistic framework rather than as experimentally established elementary reactions.

Two complementary effects of the chitinous support are considered relevant to nanoparticle formation. First, amine-containing chitinous surfaces can provide coordination-defined sites for metal-ion association and subsequent immobilization [85]. The polymer-ligand behavior of chitosan and partially deacetylated chitin has been widely exploited for metal binding and heterogeneous catalyst preparation because the availability of amino groups and the local chemical environment influence metal coordination and interfacial interactions [40,83,84,86,87]. Studies of Pd nanoparticle formation on amine-functionalized surfaces have further identified amine functionality as an important factor in surface-supported Pd nanoparticle synthesis [88].

Second, nitrogen- and oxygen-containing functional groups at the chitin interface may contribute to stabilization and spatial confinement of growing nanoparticles, thereby limiting aggregation and promoting dispersion. Chitosan has a strong affinity for transition metals and has been extensively investigated as a support for heterogeneous catalysts [84]. Often, chitin-supported metal nanoparticle catalysts outperformed cellulose-supported analogs, delivering superior catalytic performance under otherwise comparable mild reaction conditions [28,43,89,90,91]. In Pd–chitosan systems, Pd species have been shown to interact with amino groups and, depending on preparation conditions, with both amino and hydroxyl functionalities; incorporation of Pd into chitosan matrices can also enhance dispersion and reduce aggregation and leaching [83,92]. Direct precedents are provided by Pd nanoparticles supported on chitin and chitosan nanocrystals, for which dispersed Pd nanoparticles were observed on the chitinous nanocrystal surfaces and full product yield was obtained in model Heck coupling reactions under relatively mild conditions. In that study, the chitinous nanomaterial-supported catalysts compared favorably with other biomass-supported systems, including cellulose nanocrystal-supported catalysts [43].

**Scheme 2:**
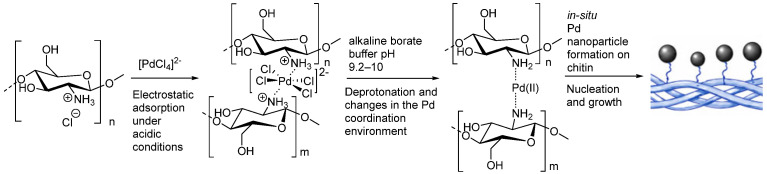
Proposed interfacial pathway for Pd nanoparticle formation on partially deacetylated chitin nanofibers. Under acidic conditions (0.1 M HCl, pH ~2), accessible surface amines are protonated and associate electrostatically with anionic ${\left[PdCl_{4}\right]}^{2-}$ species. Subsequent exposure to alkaline borate buffer (pH 9.2–10) promotes deprotonation of the amine-enriched interface and changes in Pd(II) hydrolysis/speciation. Pd nanoparticles then form in situ at the chitin interface. The detailed redox pathway leading to nanoparticle formation was not assigned experimentally.

Based on the experimental conditions used in the present study and on the literature precedents, the in situ formation of Pd nanoparticles on partially deacetylated chitin mats is proposed to involve three broadly defined stages: (1) electrostatic adsorption and ion pairing under acidic conditions, (2) deprotonation and changes in the Pd coordination environment upon transfer to alkaline medium, and (3) formation of Pd(0) nuclei followed by nanoparticle growth (Scheme 2). The precise redox process connecting Pd(II) species to Pd(0), however, remains unresolved. Therefore, these steps are presented as a plausible mechanistic framework rather than as experimentally established elementary reactions.

The partially deacetylated chitin mats were first immersed in an aqueous Na_2_PdCl_4_ solution (1 mM, pH ~2) and maintained in the dark for 72 h to promote association of Pd(II) species with the accessible nanofiber interface. Under these acidic conditions, accessible amino groups are expected to be predominantly protonated as –NH_3_^+^ [84]. Palladium sorption by chitosan under acidic conditions near pH 2 has also been reported, with the nitrogen-containing groups of chitosan playing a central role in metal uptake through mechanisms that can include ion exchange and coordination, depending on solution conditions [93].

Because the precursor was introduced as Na_2_PdCl_4_, negatively charged chloropalladate species, including [PdCl_4_]^2−^, are expected to contribute to the Pd(II) speciation during the acidic loading step. The precise distribution of Pd(II) chloro complexes depends on chloride concentration as well as pH, and a single solution species is therefore not assigned. Nevertheless, the initial uptake of Pd(II) is proposed to involve electrostatic association and ion pairing between protonated sites at the chitin interface and negatively charged Pd(II) chloro complexes.

Following the adsorption period, the mats were rinsed to remove weakly associated or unbound precursor and transferred to borate buffer at pH 9.2–10. Under these alkaline conditions, surface ammonium groups become deprotonated, increasing the population of neutral –NH_2_ donor sites. At the same time, the coordination and solution chemistry of the associated Pd(II) species are expected to change substantially. Experimental structural studies of Pd–chitosan complexes have shown that Pd can coordinate to chitosan amino groups and that increasing the pH during preparation can favor coordination environments involving both amino and hydroxyl functionalities [83]. Thus, deprotonated nitrogen donor groups, together with oxygen-containing groups at the chitin interface, may participate in retaining and stabilizing Pd(II) species during the subsequent stages of nanoparticle formation.

Transfer to alkaline medium is also expected to favor hydrolysis of Pd(II). Experimental studies of aqueous Pd(II) speciation have demonstrated the formation of hydroxide and mixed hydroxy–chloride complexes over a broad pH range, with the relative abundance of chlorinated and hydrolyzed Pd(II) species depending on pH and chloride concentration [82]. Accordingly, surface-associated chloropalladate species initially retained during the acidic loading step may undergo progressive ligand exchange and hydrolysis after transfer to pH 9.2–10. In the present system, the borate buffer is considered primarily to maintain the alkaline conditions required for deprotonation of surface ammonium groups and for changes in Pd(II) speciation and hydrolysis. Borate species present under these conditions are not assigned a direct role as reducing agents and should not be confused with borohydride-based reductants. The exact identity of the resulting surface-bound Pd(II) species cannot be established from the present data and is therefore left unspecified.

Upon prolonged exposure to the alkaline medium, the surface-associated Pd(II) species gradually undergo conversion to Pd(0)-containing nanoparticles. Experimentally, this transformation was accompanied by disappearance of the characteristic Pd(II) precursor absorption and by progressive changes in the appearance of the mats from pale yellow to brown and ultimately dark gray over the 11-day treatment period. These observations are consistent with gradual nanoparticle formation but do not, by themselves, establish the elementary redox pathway responsible for conversion of Pd(II) to Pd(0). Although nanoparticle formation occurred after transfer of the Pd-loaded mats to borate buffer, this temporal association does not demonstrate that borate is responsible for Pd(II) reduction. Rather, the buffer is proposed to establish and maintain the alkaline chemical environment in which deprotonation, ligand exchange, Pd(II) hydrolysis, and subsequent nanoparticle formation occur.

Importantly, although the literature supports Pd(II) hydrolysis, changes in Pd coordination at chitosan surfaces, and stabilization of Pd nanoparticles by amine-containing supports, it does not establish the specific reduction pathway operating under the conditions used here. No external reducing agent was deliberately added in the present procedure, and neither the borate buffer nor the chitin support is assigned a direct reducing function on the basis of the available data. The ultimate electron source responsible for net reduction of Pd(II) to Pd(0) has not been identified. Accordingly, no specific Pd(II)-to-Pd(0) reduction mechanism is assigned. The transformation is therefore described operationally as gradual conversion of surface-associated Pd(II) species into Pd(0)-containing nuclei and nanoparticles, with the underlying redox chemistry remaining unresolved.

Once the first Pd(0) nuclei are formed, the chitin surface may facilitate their retention and subsequent growth by providing localized coordination and anchoring environments. Pre-existing nuclei may in turn favor further deposition and growth of Pd in their immediate vicinity, resulting in progressive formation of nanoparticles at the accessible nanofiber interface. Literature on amine-functionalized supports and chitosan-based Pd catalysts supports the ability of such surfaces to immobilize Pd species and promote highly dispersed supported nanoparticles [43,88,92]. The slow color evolution observed over the 11-day treatment period is consistent with gradual formation and growth rather than rapid bulk precipitation. The 11-day treatment time was selected to permit slow nanoparticle formation under mild aqueous conditions and was not optimized with respect to production rate; accordingly, the present synthesis should be regarded as a proof-of-concept approach rather than an optimized manufacturing process.

Within this framework, partially deacetylated chitin is proposed to function primarily as a Pd-concentrating, coordination, anchoring, and stabilization scaffold. Surface-accessible amino groups generated by partial deacetylation increase the density of potential Pd-binding sites, while hydroxyl and residual acetamide functionalities may provide additional interfacial interactions. Because deacetylation is expected to occur preferentially in regions accessible to the treatment medium, Pd association and subsequent nanoparticle formation are proposed to be concentrated primarily at accessible regions of the nanofiber interface. This interpretation is consistent with the established affinity of chitosan-type materials for transition-metal ions and with experimental observations of Pd coordination and nanoparticle stabilization on amine-containing polysaccharide supports [43,83,84].

Overall, the observations are consistent with a pathway in which negatively charged chloropalladate species are initially concentrated at the protonated chitin interface under acidic conditions, followed by deprotonation of surface ammonium groups and changes in Pd(II) coordination and hydrolysis upon transfer to alkaline borate buffer. The borate buffer is proposed to control the alkaline reaction environment rather than to act as a chemical reducing agent. Surface-associated Pd(II) species subsequently undergo an unresolved redox transformation leading to the formation of Pd(0) nuclei, followed by their retention and progressive growth at the accessible nanofiber interface (Scheme 2). Partially deacetylated chitin is proposed to facilitate this process by concentrating Pd precursor species and providing coordination, anchoring, and stabilization sites for Pd species, nuclei, and growing nanoparticles. The precise redox pathway by which Pd(II) is converted to Pd(0) remains unresolved and would require additional time-resolved oxidation-state measurements and systematic control experiments involving variations in pH, buffer composition, atmosphere, and surface functionality.

**Figure 3:**
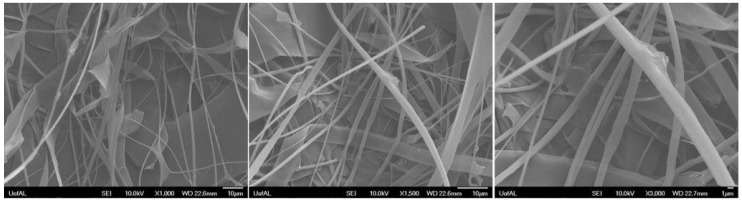
SEM micrographs of electrospun, surface-deacetylated chitin nanomats after in situ reduction of Pd(II) to Pd(0) nanoparticles. Magnifications: (**left**) ×1000, (**middle**) ×1500, (**right**) ×3000.

Scanning Electron Microscopy (SEM) analysis revealed that the electrospun, partially deacetylated chitin nanomats retained their highly fibrous and interconnected morphology after the in-situ reduction of Pd(II) to Pd(0) (Figure 3). No evidence of fiber fusion, collapse, or agglomeration was observed across magnifications (×1000–×3000), confirming that the reduction process and subsequent nanoparticle immobilization did not compromise the structural integrity of the chitin network. Individual Pd nanoparticles were not directly discernible by SEM due to their expected sub-10 nm size, which is below the instrument’s resolution limit; however, the smooth and continuous fiber surfaces suggest uniform nanoscale metal deposition, consistent with the spectroscopic findings discussed in the following sections.

### 3.3. Extension of the Proposed Ion-Exchange Mechanism to Au, Ag, and Co-Loaded Systems

The same general loading strategy was extended to Au and Ag systems. Surface-modified chitin mats were equilibrated with aqueous KAuCl_4_ or AgNO_3_ precursor solutions at approximately pH 2 for 72 h in the dark. Under these acidic conditions, accessible amino groups are expected to be predominantly protonated. For Au(III), electrostatic association between protonated interfacial sites and anionic chlorometallate species is therefore plausible during the initial loading step. Ag(I) speciation and binding under these conditions may involve different interactions and is not assigned to a single interfacial mechanism.

Following transfer to alkaline borate buffer, deprotonation of accessible amino groups and changes in metal speciation may permit stronger coordination to nitrogen- and oxygen-containing sites and facilitate subsequent nanoparticle formation, supported by the characteristic ruby-red coloration. Confinement of metal ions to discrete coordination sites on the partially deacetylated chitin suppressed aggregation and produced uniformly dispersed nanoparticles firmly anchored to the biopolymer surface.

For co-loaded Pd–Au systems, equimolar mixtures of Na_2_PdCl_4_ and KAuCl_4_ (0.5/0.5 mM, pH~2) were used to promote co-adsorption and potential alloy formation. The subsequent ion-exchange and nanoparticle formation in alkaline borate buffer produced Pd/Au-containing nanomats with nanoscale metal-containing features. This result highlights the versatility of the immobilization and reduction mechanism across both mono- and co-loaded systems.

### 3.4. Characterization

#### 3.4.1. UV-Vis Spectroscopy

**Figure 4:**
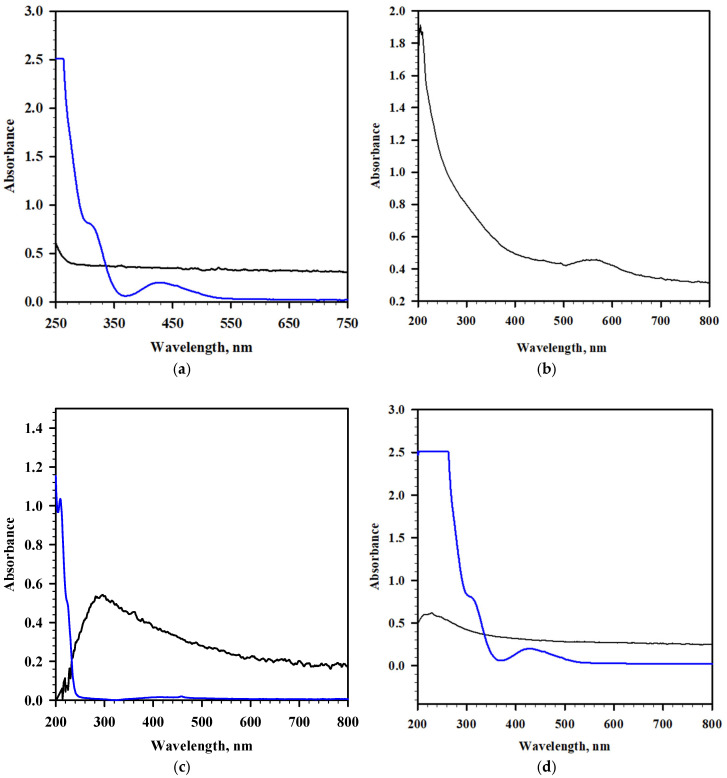
UV–Vis spectra of metal–chitin nanomats and their corresponding metal-ion precursors following borate-buffer reduction: (**a**) Pd–chitin mat (black) compared with the Pd^2+^ precursor solution (blue); (**b**) Au–chitin mat (black); (**c**) Ag–chitin mat (black) compared with the Ag^+^ precursor (blue); and (**d**) co-loaded Pd–Au chitin mat (black) compared with the Pd^2+^ precursor (blue).

UV–Vis spectroscopy was used to follow disappearance of the characteristic Pd(II) precursor absorption during nanoparticle formation. The aqueous Na_2_PdCl_4_ precursor exhibited a characteristic absorption band centered at ~430 nm, corresponding to ligand-to-metal charge transfer transitions in [PdCl_4_]^2−^ complexes [94,95]. After the treatment with borate buffer (pH 10), this band completely disappeared in the Pd–chitin composite spectrum (Figure 4a). Together with STEM–EDX/HRTEM characterization, these changes are consistent with formation of Pd-containing nanoparticles including Pd(0) domains, consistent with previous reports for Pd systems [94,95].

The UV–Vis spectrum of KAuCl_4_ solution displayed an intense absorption band in the 290–320 nm region, characteristic of [AuCl_4_ complexes. After nanoparticle formation in alkaline borate buffer, this peak disappeared and a new, broad surface plasmon resonance (SPR) band at ~540 nm appeared, supporting the formation of metallic Au-containing nanoparticles [96] on the chitin surface (Figure 4b). The plasmonic band intensity and position suggested the presence of well-dispersed spherical Au^0^ NPs smaller than 10 nm. The absence of additional peaks indicates uniform size distribution and minimal aggregation.

The AgNO_3_ precursor solution exhibited the expected absorption band associated with Ag^+^ ions at 215–220 nm. Upon borate-buffer-mediated reduction, a new broad absorption feature appeared at ~300 nm (Figure 4c), while no classical visible-region SPR band (400–450 nm) was observed. The Ag SPR is strongly size-dependent and can collapse entirely when nanoparticle diameters shrink to only a few nanometers. In such ultrasmall domains, the optical response is no longer dominated by collective conduction-electron oscillations; instead, the UV–Vis spectrum becomes governed by interband transitions and ligand–metal charge-transfer bands, which typically occur in the 250–320 nm region [97]. This behavior is fully consistent with our results: the spectrum displays a single broad band at ~300 nm with no detectable SPR, even though STEM imaging confirms the presence of ultrasmall Ag-containing nanoparticles. However, definitive assignment of the Ag oxidation state would require additional surface-sensitive or diffraction-based analysis. The disappearance or damping of the SPR is consistent with extremely small Ag-containing nanoparticles uniformly dispersed across the chitin surface.

The co-loaded Pd–Au system exhibited spectral features distinct from those of the monometallic samples. The Pd^II^ precursor showed a characteristic peak at ~430 nm, which disappeared completely after reduction at pH 10, confirming nanoparticle formation (Figure 4d). However, no distinct Au plasmon-resonance band near ~520–530 nm was observable in the UV–vis spectra of the reduced composite. This absence is consistent with close Pd–Au association in nanoparticles [98].

After reduction, the Pd–Au nanocomposite retained a broad UV absorption band at ~220 nm. This band is characteristic of metallic Pd- and Au-based nanoparticles and originates from d→sp interband electronic transitions. In Pd–Au-containing nanoparticles, suppression or damping of the Au visible SPR can make the deep-UV interband transition the dominant spectral feature following reduction [99,100].

#### 3.4.2. Elemental Mapping

Elemental distribution within the metal–chitin nanocomposites was examined by scanning transmission electron microscopy coupled with energy-dispersive X-ray spectroscopy (STEM–EDX). The use of STEM–EDX, rather than conventional TEM–EDX, provided high-resolution elemental mapping, allowing direct correlation between nanoparticle morphology and metal distribution on the chitin nanofiber surface.

**Figure 5:**
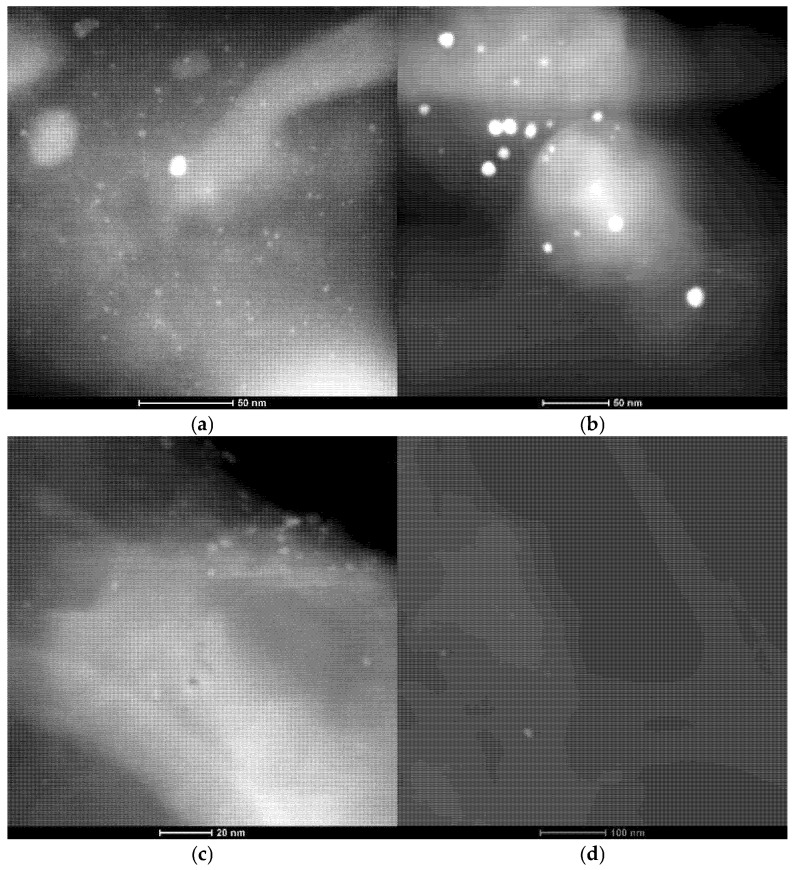
(**a**–**d**). STEM images of IL-processed chitin nanomats showing uniformly distributed metal nanoparticles on nanofiber surfaces: (**a**) Pd (3.2 ± 1.0 nm), (**b**) Au (6.4 ± 2.6 nm), (**c**) Ag (2.6 ± 1.2 nm), and (**d**) co-loaded Pd–Au, showing nanoscale particles distributed along the nanofiber surfaces. The examined STEM regions show nanoscale, approximately spherical metal-containing particles distributed along the chitin nanofiber surfaces, with no large aggregates observed in the imaged areas.

Bright-field STEM images revealed that Pd nanoparticles with near spherical morphology were formed on the surface of deacetylated chitin mats and distributed along the nanofiber surfaces (Figure 5a and Figure S8). Image-based analysis of Pd nanoparticles from the examined STEM regions gave a mean measured diameter of 3.2 ± 1.0 nm (Table S2 and Figure S9). No aggregates larger than 10 nm were observed in the analyzed regions, indicating nanoscale dispersion under the conditions examined. Comparable size control is typically achieved only through seed-mediated synthetic strategies that confine growth within the sub-10 nm range. Consistent observations have been reported for other amine-bearing supports in borate-based media: biotemplated Pd deposition on amine-rich plant viruses (TMV, BSMV) in borate buffer produced 3–5 nm nanoparticles anchored to lysine residues [65], while in chitosan matrices, borate buffer gently deprotonated –NH_3_^+^ groups and moderated Pd reduction to 2–6 nm particles [101] without polymer degradation or aggregation. Likewise, amine-functionalized SBA-15 mesoporous silica functionalized with –NH_2_ yielded ~3–5 nm AgPd bimetallic nanoparticles templated within mesopores under similar conditions [102].

The Au–chitin nanomats exhibited a similarly homogeneous dispersion of bright, approximately spherical nanoparticles decorating the fiber surfaces (Figure 5b and Figure S10). STEM–EDS confirmed the presence of Au within these nanoparticles. In addition, disappearance of the characteristic [AuCl_4_]^−^ precursor absorption and emergence of a broad surface plasmon resonance (SPR) band centered at approximately 540 nm are consistent with the formation of Au(0)-containing nanoparticles immobilized on the chitin support. The mean particle diameter was 6.4 ± 2.6 nm (Table S3 and Figure S11), slightly larger than that observed for the Pd-containing nanoparticles. The absence of fused clusters or large aggregates further indicates that the chitin matrix promotes spatial separation and dispersion of the Au-containing nanoparticles.

For the Ag–chitin nanomats, uniformly distributed nanoparticles were also observed across the nanofiber surfaces (Figure 5c and Figure S12). The high-contrast regions are consistent with Ag-containing nanoparticles formed following treatment in alkaline borate buffer. The average particle diameter was 2.6 ± 1.2 nm (Table S4 and Figure S13), smaller than those observed for the Pd- and Au-containing systems. The observed particle sizes and absence of extensive aggregation within the examined regions are consistent with stabilization and spatial confinement of the nanoparticles by the chitin matrix.

High-resolution STEM imaging of the co-loaded Pd–Au chitin nanomats revealed approximately spherical nanoscale particles distributed along the chitin fiber surfaces (Figure 5d and Figure S14). Because a sufficiently large particle-by-particle dataset was not available for this sample, a statistically representative mean particle diameter is not assigned.

The EDS spectrum of the Pd–chitin nanomats confirmed the incorporation of palladium within the nanostructured composite. The most intense emission peaks correspond to the O Kα (~0.53 keV) [103,104] and Pd L α /L β (~2.84–2.99 keV) [105] transitions (Figure S15), characteristic of oxygen and palladium, respectively. The oxygen signal primarily originates from the polysaccharide framework, namely, hydroxyl and residual carbonyl groups within the chitin backbone, as well as trace contributions from surface-bound borate species or adsorbed hydroxyls.

The palladium L-series peaks, Lα (corresponding to the electronic transition L_3_ → M_5_, photon energy of ~2838 eV) and Lβ_1_ (corresponding to the electronic transition M_4_ → L_3_ (3d_3_/_2_ → 2p_3_/_2_), centered near 2.8–3.0 keV), arise from L-shell electronic transitions and provide the most reliable fingerprint for Pd in thin, organic-based matrices analyzed under moderate accelerating voltages (15–30 kV) [106]. Although Pd also exhibits K-series lines at higher energies (~21 keV), these transitions are relatively weak under standard EDS conditions and often fall near the upper sensitivity limit of the silicon drift detector. This consideration is particularly important for biopolymer-supported samples such as chitin, where low-atomic-number elements increase X-ray absorption and scattering, thereby reducing the efficiency of high-energy X-ray detection. Consequently, the Pd L-series lines were preferentially used for both qualitative and quantitative identification, providing consistent compositional data across multiple sampling regions. Quantitative EDS analysis yielded an O:Pd weight ratio of 42.18:57.82 wt%, confirming that palladium is the predominant heavy element and validating the successful in situ nanoparticle formation and anchoring on the chitin surface.

The EDS spectrum of the Au–chitin nanomats (Figure S16) displays the characteristic Au peaks at 2.12 keV (Mα) and 9.71 keV (Lα_1_), together with a minor O Kα peak at 0.52 keV, in agreement with previously reported Au nanoparticle spectra [107,108]. The most intense peaks correspond to the O K and Au L emission lines, characteristic of oxygen from the chitin framework and metallic gold, respectively. Additional minor signals arise from carbon (C K) and nitrogen (N K) in the biopolymer matrix. The quantitative analysis yielded an O:Au weight ratio of 94.73:5.27 wt%, indicating moderate gold loading consistent with the surface-deposited nanoparticles observed in the STEM images. These combined STEM and EDS results demonstrate that gold nanoparticles were uniformly anchored to the chitin nanofiber surface without detectable aggregation, validating the effectiveness of the IL-processed chitin scaffold as a stabilizing and nucleation-supporting matrix for noble metal nanoparticle growth.

Meaningful EDX spectra could not be obtained for the Ag–chitin nanomats. The silver nanoparticles were the smallest in the series (2.6 ± 1.2 nm) and uniformly dispersed across thin chitin fibers, resulting in weak signal intensity relative to the surrounding biopolymer matrix. The inherently low atomic number and low X-ray yield of Ag further reduced detection sensitivity under the low-voltage STEM-EDX settings required to minimize beam damage to the chitin support. Prolonged exposure caused local electron-beam heating and partial migration of the Ag nanoparticles, precluding reliable quantification. Nevertheless, the strong bright-field contrast and uniform nanoparticle distribution observed in the STEM images provide clear morphological evidence for the formation of Ag-containing nanoparticles on the chitin surface.

HRTEM analysis of the Pd–Au co-loaded chitin nanomats revealed nanoscale, electron-dense particles associated with the chitin nanofiber matrix. FFT analysis of selected particles yielded a dominant lattice spacing of approximately 0.23 nm, which lies between the reported fcc Au(111) (~0.235 nm) and Pd(111) (~0.224 nm) spacings. This observation, together with suppression of a distinct Au surface-plasmon band in the UV–Vis spectrum, is consistent with possible close association of Pd- and Au-containing species. However, these measurements do not distinguish among alloyed particles, core–shell or heterostructured arrangements, closely contacted monometallic domains, or separate Pd- and Au-containing nanoparticle populations. Because element-resolved mapping and an independently determined Pd:Au ratio were not available, the precise compositional architecture of the material remains unresolved. Accordingly, the sample is described here as a Pd–Au co-loaded chitin nanomat, without assignment of a specific bimetallic nanoparticle structure (Figure S17).

### 3.5. Performance Testing

**Scheme 3:**
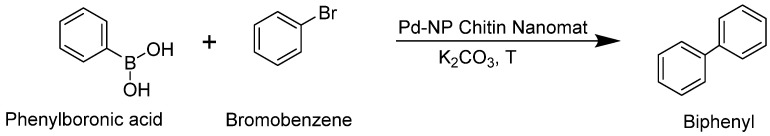
Suzuki coupling reaction between bromobenzene and phenylboronic acid.

Au, Ag, and Pd–Au systems were included to demonstrate generality of metal immobilization, whereas catalytic evaluation was focused on Pd because Suzuki–Miyaura coupling is Pd-mediated. The catalytic activity of the Pd–chitin nanomats was evaluated using the Suzuki–Miyaura cross-coupling reaction between bromobenzene and phenylboronic acid under static conditions to produce biphenyl as a model product (Scheme 3). This benchmark reaction was selected to assess the accessibility, stability, and reusability of immobilized Pd nanoparticle sites under mild conditions. Reactions were conducted in a 2:1 (*v*/*v*) ethanol/water mixture at 70 °C for 12 h. The mixed solvent provided both solubility for the organic substrates and wetting of the hydrophilic chitin matrix, ensuring efficient diffusion to immobilized Pd sites.

#### 3.5.1. Catalyst Loading Study

The catalyst loading ranged from 0.0019–0.017 mol % Pd relative to bromobenzene. To determine the minimum active catalyst requirement, Suzuki–Miyaura coupling reactions containing 1 equiv. bromobenzene (0.50 mmol), 1.5 equiv. phenylboronic acid (0.75 mmol), and 2 equiv. K_2_CO_3_ (1.00 mmol) were performed under identical conditions without stirring, thereby imposing limited mass-transfer conditions. Catalyst (Pd–chitin mat) masses of 0.048, 0.114, 0.321, and 0.465 mg (equivalent to 1.0, 2.3, 6.4, and 9.2 µg Pd) were evaluated. As shown in Table 1, quantitative conversion was achieved using 6.4–9.2 µg Pd (0.012–0.017 mol %), highlighting the high apparent activity of the immobilized Pd nanoparticles. Under the static conditions employed, Pd–chitin nanomats containing the higher catalyst loadings produced >99% conversion and >99% selectivity. Because the reactions were conducted without stirring and the catalyst was used as a free-standing fibrous mat, the observed conversions may reflect contributions from external and internal mass-transfer limitations. Accordingly, these results are interpreted as apparent catalytic performance under the specified reaction conditions rather than as measurements of intrinsic catalytic kinetics.

Control experiments using pristine chitin mats produced no detectable biphenyl under the reaction conditions, demonstrating that the chitin support alone does not measurably promote the Suzuki–Miyaura coupling. The performance of the Pd–chitin nanomats is compared separately with previously reported cellulose- and chitosan-supported Pd catalytic systems in Table 2.

Product analysis by gas chromatography–mass spectrometry (GC–MS) confirmed exclusive formation of biphenyl, with no detectable by-products such as phenol, biphenyl oxide, or dehalogenated benzene, indicating a clean and selective catalytic pathway.

When benchmarked against reported cellulose- and chitosan-supported Pd catalysts for Suzuki and Heck coupling reactions (Table 2), it becomes clear that across cellulose- and chitosan-based heterogeneous Pd catalysts, catalytic performance is generally achieved at the expense of relatively high Pd loadings, limited nanostructural control, or incomplete reporting of selectivity and conversion. Cellulose nanofiber, nanosponge, and nanocrystal supports typically require 0.1–1.0 mol % Pd to reach high activity, with conversion values below quantitative levels despite excellent selectivity. Chitosan-based systems, while benefiting from metal coordination through amine functionalities, frequently rely on bulk matrices, beads, or flakes, necessitating higher Pd loadings (0.17–2.0 mol %) and often reporting performance solely in terms of isolated yield rather than explicit selectivity or conversion. Notably, the only reported example of a nanofibrillated chitosan support employs electrospun, glutaraldehyde-crosslinked chitosan nanofibers bearing coordinated Pd(II) species for Heck coupling, achieving high activity but still requiring substantially higher Pd loadings and lacking nanoscale control over surface-localized Pd(0) domains.

In contrast, the partially deacetylated chitin–Pd electrospun nanomats reported here uniquely combine a nanofibrous, free-standing architecture with amine-enriched accessible interface, enabling quantitative conversion and >99% selectivity at Pd loadings as low as 0.0019–0.017 mol %, competitive with or lower than many reported cellulose- and chitosan-supported Pd systems. This unprecedented efficiency arises from the synergistic effects of nanoscale confinement, surface-localized coordination sites, and uniform Pd dispersion, establishing these chitin nanomats as one of the most Pd-efficient, selective, and structurally robust biopolymer-supported catalysts reported to date.

#### 3.5.2. Catalyst Recyclability and Stability

Recyclability experiments were conducted on Pd–chitin nanomats to evaluate short-term operational stability under repeated Suzuki–Miyaura coupling conditions. Because the catalyst is a free-standing nanomat that must be physically recovered, rinsed, dried, and re-weighed between runs, recyclability testing was limited to three (3) cycles; beyond this point, minor mechanical damage and handling-related mass loss introduce uncertainty in normalizing catalytic activity by catalyst mass.

The freshly prepared Pd–chitin nanomats achieved quantitative conversion of 1-bromobenzene with exclusive formation of biphenyl in the first and second cycles (Table 3, and Figure S18), indicating that the immobilized Pd remains catalytically competent over consecutive runs. Upon extension to a third cycle, conversion decreased from >99% to 85%, while selectivity remained >99%, indicating partial loss of apparent catalytic activity without a measurable decrease in product selectivity. Several factors may contribute to this decline, including physical loss of nanomat material during recovery and handling, Pd leaching, nanoparticle growth or aggregation, surface poisoning, and changes in the oxidation state or coordination environment of surface-accessible Pd species.

Based on the gravimetrically estimated Pd loading and the measured conversions, apparent TON values ranged from approximately 5700 to 15,400, corresponding to average 12-h TOF values of approximately 480–1290 h^−1^. These values are provided as apparent performance metrics rather than intrinsic kinetic parameters.

For comparison, additional experiments were conducted using Pd–chitin samples stored for 1 month under ambient conditions prior to catalysis. These samples exhibited slightly lower initial conversion (96%) and more pronounced activity loss upon reuse relative to freshly prepared Pd–chitin nanomats, while maintaining ≥99% selectivity. This behavior indicates reduced effective activity of surface-accessible Pd sites after storage, without alteration of the reaction pathway.

Separately, co-loaded Pd–Au chitin nanomats showed lower initial conversion (~86%) than the monometallic Pd–chitin nanomats while retaining >99% selectivity (Figure S19). Because the relative Pd and Au contents and their nanoscale spatial arrangement were not independently determined, the origin of the lower activity cannot be assigned unambiguously; it may reflect differences in accessible Pd content and/or local Pd–Au interactions.

## 4. Conclusions

IL processing of chitin, coupled with partial deacetylation of the accessible nanofiber interface, provides a versatile route to engineer renewable, nanofibrous biopolymer supports. By dissolving high-molecular-weight chitin, sourced from shrimp-shell waste, in [C_2_C_1_im][OAc] and electrospinning it into free-standing electrospun nanofibrous mats (20–25 nm), we create robust, highly porous nanofibrous catalyst support (rather than persistent inorganic/carbon supports) subsequently activated by surface deacetylation while preserving the mechanically stable α-chitin core. The tailored support enables association and immobilization of Pd(II), Au(III), Ag(I), and mixed Pd(II)/Au(III) precursors at the modified chitin interface, followed by changes in metal coordination and speciation under alkaline conditions and formation of surface-associated metal-containing nanoparticles.

UV–Vis, STEM–EDX, and HRTEM confirmed formation of nanoscale metal-containing particles. Image-based STEM measurements gave mean particle diameters of 3.2 ± 1.0 nm for Pd, 6.4 ± 2.6 nm for Au, and 2.6 ± 1.2 nm for Ag; the co-loaded Pd–Au sample exhibited nanoscale, well-dispersed particles within the examined regions. The principal performance advantage of the present Pd–chitin nanomats is not the attainment of high conversion alone, since comparable conversions have been reported for several cellulose- and chitosan-supported Pd catalysts. Rather, the present system achieves high apparent catalytic performance at substantially lower estimated Pd loadings than many of the biopolymer-supported systems summarized in Table 2. In addition, the catalyst is presented as a free-standing nanofibrous mat that can be physically recovered after reaction. Accordingly, the key advantages of the present architecture are low Pd utilization, accessible nanofibrous morphology, and recoverability of the catalyst format, rather than uniquely high conversion. The Pd–chitin nanomats exhibited high apparent catalytic performance at low estimated Pd loadings, with >99% conversion and >99% selectivity achieved at estimated Pd loadings of 0.0120–0.0173 mol% under the conditions examined. Recyclability tests show that conversion decreased from >99% in the first two cycles to 85% in the third cycle, while selectivity remained >99%. The present recyclability data do not distinguish among possible deactivation pathways, including physical catalyst loss, Pd leaching, nanoparticle growth or aggregation, surface poisoning, and changes in Pd oxidation state or coordination environment.

The high apparent catalytic performance of the Pd–chitin nanomats is consistent with the combination of nanoscale Pd dispersion, accessible interfacial anchoring sites, and the open nanofibrous morphology of the support. The modified chitin interface may stabilize Pd-containing nanoparticles through interactions with amino and hydroxyl functionalities, while the absence of added surfactant stabilizers may help maintain accessible catalytic surfaces. Because the reactions were conducted under static conditions, however, the relative contributions of intrinsic catalytic activity and external or internal mass-transfer effects were not independently determined.

The high activity of the Pd–chitin nanomats is attributed to the combination of nanoscale Pd dispersion, accessible interfacial anchoring sites, and the open nanofibrous morphology of the chitin support. The modified chitin interface likely stabilizes Pd nanoparticles through interactions with amino and hydroxyl groups, while the absence of added surfactant stabilizers helps maintain accessible catalytic surfaces. Although additional leaching, XPS, and post-reaction microscopy studies would be needed to fully define the active-site structure and deactivation pathway, the available data demonstrate that the Pd–chitin nanomats provide highly efficient Suzuki–Miyaura coupling under low-Pd-loading conditions. Future work could build on this proof-of-concept by systematically examining the effects of pH, surface amine functionality, Pd speciation, and reaction conditions on metal uptake and nanoparticle formation. Time-resolved characterization of Pd oxidation state would also help clarify the unresolved Pd(II)-to-Pd(0) conversion and distinguish among possible mechanistic pathways.

The sustainability implications of the present system should be considered in the context of the complete preparation and catalytic workflow. Although the support is derived from shrimp-shell waste and nanoparticle formation is conducted in aqueous borate medium without an added molecular reducing agent or surfactant stabilizer, the current laboratory-scale process also involves ionic-liquid processing, repeated aqueous coagulation and washing, alkaline surface treatment, prolonged metal loading and nanoparticle-formation steps, ethanol use, and noble-metal precursors. The 11-day nanoparticle-formation period is conducted at ambient temperature but has not been optimized with respect to process time or throughput. In addition, recovery and reuse of [C_2_C_1_im][OAc] were not quantified in the present study. Dichloromethane is not used in catalyst preparation but is employed during post-reaction extraction and GC–MS sample preparation. Therefore, the present study demonstrates a renewable biopolymer-based catalyst-support concept rather than an optimized green manufacturing process. A rigorous sustainability comparison will require complete mass and energy balances, including PMI and E-factor analysis, solvent and ionic-liquid recovery efficiencies, and energy requirements after process optimization.

## Figures and Tables

**Table 1:** Catalyst loading study for the Suzuki–Miyaura coupling of bromobenzene (0.5 mmol) with phenylboronic acid (0.75 mmol) using K_2_CO_3_ (1 mmol) in EtOH/H_2_O (2:1 *v*/*v*), 70 °C, 12 h.

Catalyst	Mass of Pd–Chitin Mat (mg, dry)	Estimated Pd Content (µg)	Pd (mol%) ^a^	Selectivity (%)	Conversion (%)	Apparent TON ^c^	Average TOF (h^−1^) ^d^
Pd–chitin	0.048	1.0	0.0019	>99	29	~15,431	~1286
Pd–chitin	0.114	2.3	0.0043	>99	62	~14,344	~1195
Pd–chitin	0.321	6.4	0.0120	>99	>99	>8231	>686
Pd–chitin	0.465	9.2	0.0173	>99	>99	>5726	>477
Chitin	0.500	0.0	0.0000	ND ^b^	ND ^b^	ND ^b^	ND ^b^

^a^ Relative to limiting reagent. ^b^ ND: Product not detected. ^c^ TON was calculated as moles of bromobenzene converted to biphenyl per mole of gravimetrically estimated Pd. ^d^ Average TOF was calculated as TON divided by the 12 h reaction time. Because Pd loading was determined gravimetrically, the reported TON and TOF values are considered apparent performance metrics. For entries reported as >99% conversion, TON and TOF are presented as lower-bound values calculated using 99% conversion.

**Table 2:** Benchmark Comparison of Cellulose- and Chitosan-Supported Pd Catalysts for Suzuki–Miyaura and Heck Coupling Reactions.

Coupling	Catalyst Support	Support Type	Mol% Catalyst	Selectivity (%)	Conversion (%)	Key Observations	Ref.
Heck	Bacterial cellulose	Nano-fibers	0.10	NR	80–97	High activity, one of the lowest Pd loading among reported systems	[109]
Suzuki	Cellulose	Nano-sponge	0.80	>99	96	Improved dispersion vs native cellulose	[110]
Suzuki	Cellulose	Nano-crystals	0.50–1.00	>99	95–99	High activity; Pd stabilized mainly by –OH groups	[28]
Heck	Chitosan	Flakes	0.35	NR	97–99	High activity; Pd stabilized by –NH_2_ groups; > 9 cycles. Not nanostructured support	[78]
Heck	Chitosan	Cross-linked nano-fibers	0.17	NR ^a^	>99	Coordinated Pd(II) species; catalytic activity attributed to polymer–metal complexation; limited nanoscale control over metal speciation	[51]
Suzuki	Chitosan	Beads	2.00	NR ^a^	91 (best)	Relatively high catalyst loadings, no nanostructure	[111]
Suzuki	Chitosan	Bulk	0.020	NR ^a^	NR ^a,b^	Explicit selectivity metrics were not provided; performance evaluated based on product yield	[112]
Suzuki	Surface-de-acetylated chitin	Nano-fibers	0.0012–0.017	>99	>99	Engineered, surface-anchored Pd(0) NP catalyst; Quantitative conversion at ≥10× lower Pd loading vs reported cellulose/chitosan system	This work

^a^ Selectivity metrics were not explicitly reported; ^b^ reaction performance was evaluated primarily based on product yield (85%).

**Table 3:** Conversion and selectivity for the Suzuki coupling catalyzed by Pd–chitin nanomats.

Catalyst	Pd (mol% Catalyst)	Cycle	Conversion of 1-Bromobenzene (%)	Selectivity to Biphenyl (%)
Pd–chitin	0.017	1st	>99	>99
		2nd	>99	>99
		3rd	85.0	>99

## Data Availability

The raw data supporting the conclusions of this article will be made available by the authors on request.

## Supplementary Materials

The following supporting information can be downloaded at: https://www.mdpi.com/article/10.3390/polym18151872/s1, Figure S1: 13C CP-MAS SS NMR spectrum of chitin extracted from shrimp shell biomass; Figure S2: Powder X-ray diffraction (pXRD) pattern of the IL-extracted chitin-based material; Figure S3: Fourier transform infrared (FTIR) spectrum of IL-extracted chitin; Figure S4. 13C CP-MAS SS NMR spectrum of the prepared chitin nanomat; Figure S5. Powder X-ray diffraction (pXRD) pattern of the as-produced chitin nanomat prepared from IL-extracted chitin-based material; Figure S6. Fourier transform infrared (FTIR) spectrum of the prepared chitin nanomat; Table S1. Mercury intrusion porosimetry parameters of representative electrospun chitin nanomats; Figure S7. Mercury intrusion porosimetry pore-size distributions of representative electrospun chitin nanomats; Figure S8. Additional STEM images of IL-processed chitin nanomats showing uniformly distributed Pd nanoparticles on nanofiber surfaces; Table S2. Image-based dimensional measurements of Pd nanoparticles (n = 20); Figure S9. Distribution of image-based diameter measurements for Pd nanoparticles (n = 20); Figure S10. Additional STEM images of IL-processed chitin nanomats showing uniformly distributed Au nanoparticles on nanofiber surfaces; Table S3. Image-based dimensional measurements of Au nanoparticles (n = 24); Figure S11. Distribution of image-based diameter measurements for Au nanoparticles (n = 24); Figure S12. Additional STEM images of IL-processed chitin nanomats showing uniformly distributed Ag nanoparticles on nanofiber surfaces; Table S4. Image-based dimensional measurements of Ag nanoparticles (n = 30); Figure S13. Distribution of image-based diameter measurements for Ag nanoparticles (n = 30). Histogram bin width = 0.75 nm; Figure S14. Additional STEM images of IL-processed chitin nanomats showing uniformly distributed Pd–Au nanoparticles on nanofiber surfaces; Figure S15. EDS spectrum of Pd–chitin nanomats; Figure S16. EDS spectrum of Au–chitin nanomats; Figure S17. Low-magnification TEM overview of the Au–Pd–chitin nanomats showing the ultrathin chitin fiber network and the distribution of electron-dense bimetallic nanoparticles across the film; High-resolution TEM micrograph of an individual Au–Pd nanoparticle anchored on the chitin surface, highlighting its spherical morphology and crystalline contrast; Figure S18. GC–MS chromatogram after the Suzuki–Miyaura reaction catalyzed by Pd–chitin nanomats; Figure S19. GC–MS chromatogram after the Suzuki–Miyaura reaction catalyzed by Pd–Au–chitin nanomats.
