# Supplementary material for: Ionic-Liquid-Engineered Chitin Nanofiber Supports for Low-Loading Pd Catalysts
Source: Polymers (Basel). 2026 Jul 30;18(15):1872. doi: 10.3390/polym18151872 (PMC13468210; doi:10.3390/polym18151872)
Supplement: Supplementary file 1 [file polymers-18-01872-s001.zip › polymers-4430292-supplementary.pdf]

Ionic-Liquid–Engineered Chitin Nanofiber Supports for Low-Loading Pd Catalysts

Oleksandra Zavgorodnya,[a] Rajkumar Kore,[a] Julia L Shamshina,[b,c*] and Robin D. Rogers [a,d,*]

Oleksandra Zavgorodnya,[a] Hemant Choudhary,[b] Rajkumar Kore,[a] Julia L Shamshina[c,d*] and Robin D. Rogers [a,e*]

[a] Department of Chemistry and Biochemistry, The University of Alabama, Tuscaloosa, Alabama 35487, USA
[b] Department of Bioresource and Environmental Security, Sandia National Laboratories, Livermore, CA 94551, USA
[c] Fiber and Biopolymer Research Institute (FBRI), Texas Tech University, Lubbock, TX 79409, USA
[d] Department of Chemistry and Biochemistry, Texas Tech University, Lubbock, TX 79409, USA
[e] 525 Solutions, Inc., PO Box 2206, Tuscaloosa, AL 35403, USA

Corresponding: JShamshi@TTU.edu; Robin.Rogers@525Solutions.com

Table of Contents

1. Characterization of Starting Material (Chitin Polymer Extracted from Biomass) S3
1.1. ^{13}C CP-MAS SS NMR S3
1.2. pXRD Diffractogram S4
1.3. Fourier Transform Infra-Red Spectrum S5
2. Characterization of Chitin Nanomat Obtained by Electrospinning S6
2.1. ^{13}C CP-MAS SS NMR S6
2.2. pXRD Diffractogram S7
2.3. Fourier Transform Infra-Red Spectrum S8
2.4. Mercury Intrusion Porosimetry Data S9
3. Additional STEM Images of Metal Nanoparticles and Image-Based Dimensional Measurements S11
3.1. Palladium S11
3.2. Gold S12
3.3. Silver S14

3.4. Palladium-Gold S16

4. Energy-Dispersive X-ray Spectroscopy (EDS) S17

4.1. Palladium S17

4.2. Gold S18

5. TEM Data for the Au–Pd–Chitin Nanomat S19

6. Gas Chromatography–Mass Spectrometry (GC–MS) Results S20

1. Characterization of Starting Material (Chitin Polymer Extracted from Biomass)

1.1. ^{13}C CP-MAS SS NMR

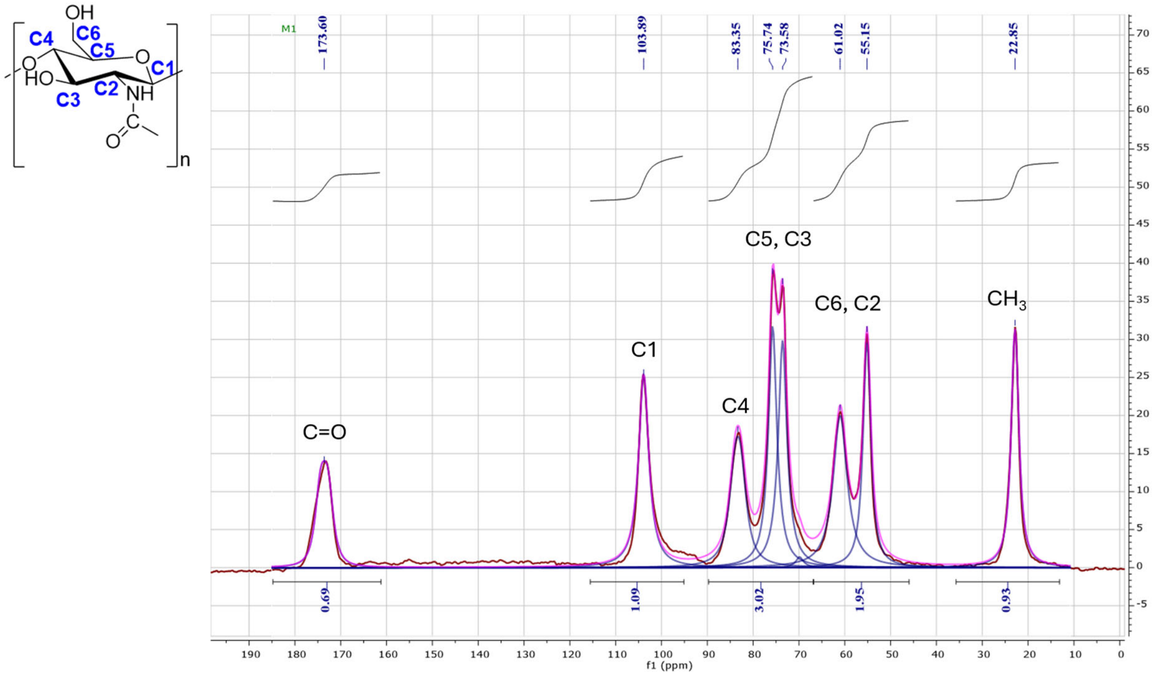

Figure S1. ^{13}C CP-MAS SS NMR of the chitin extracted from shrimp shell biomass. ^{13}C NMR peak list (δ, ppm → assignment): ~173.60 ppm → C=O (amide / acetyl carbonyl); ~103.89 ppm → C1 (anomeric carbon); ~83.35 ppm → C4; ~75.74 ppm → C5; ~73.58 ppm → C3; ~61.02 ppm → C6 (CH_2–OH); ~55.15 ppm → C2 (C–NHAc); ~22.85 ppm → CH_3 (acetyl methyl). Integrals (as reported in the spectrum): 0.69 (C=O), 1.09 (C1), 3.02 (C4+C5+C3), 1.95 (C6+C2), 0.93 (CH_3). (It should be noted that carbonyl signal intensities in ^{13}C CP/MAS are known to be non-quantitative; therefore, integral ratios involving the C=O resonance should be interpreted qualitatively.)

1.2. pXRD Diffractogram

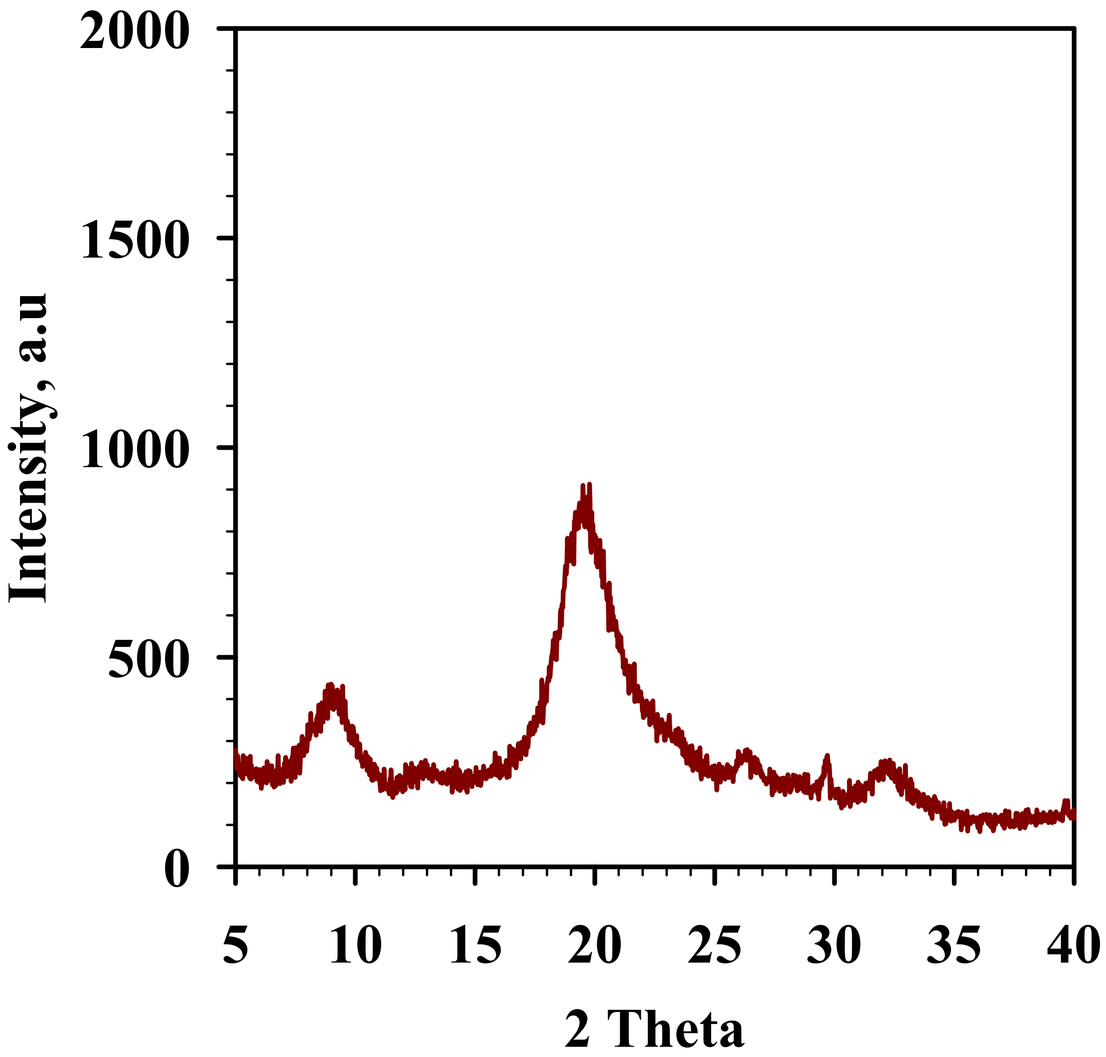

Figure S2. Powder X-ray diffraction (pXRD) pattern of the IL-extracted chitin–based material. Diffraction maxima are observed at **2θ = 9.22°, 12.84°, 19.44°, and 26.22°**, which are characteristic of the polymer with reduced crystallinity obtained via ionic-liquid processing relative to native chitin polymer. The broadened reflections and reduced long-range order are consistent with disruption of the native hydrogen-bonding network during IL dissolution and regeneration

1.3. Fourier Transform Infra-Red Spectrum

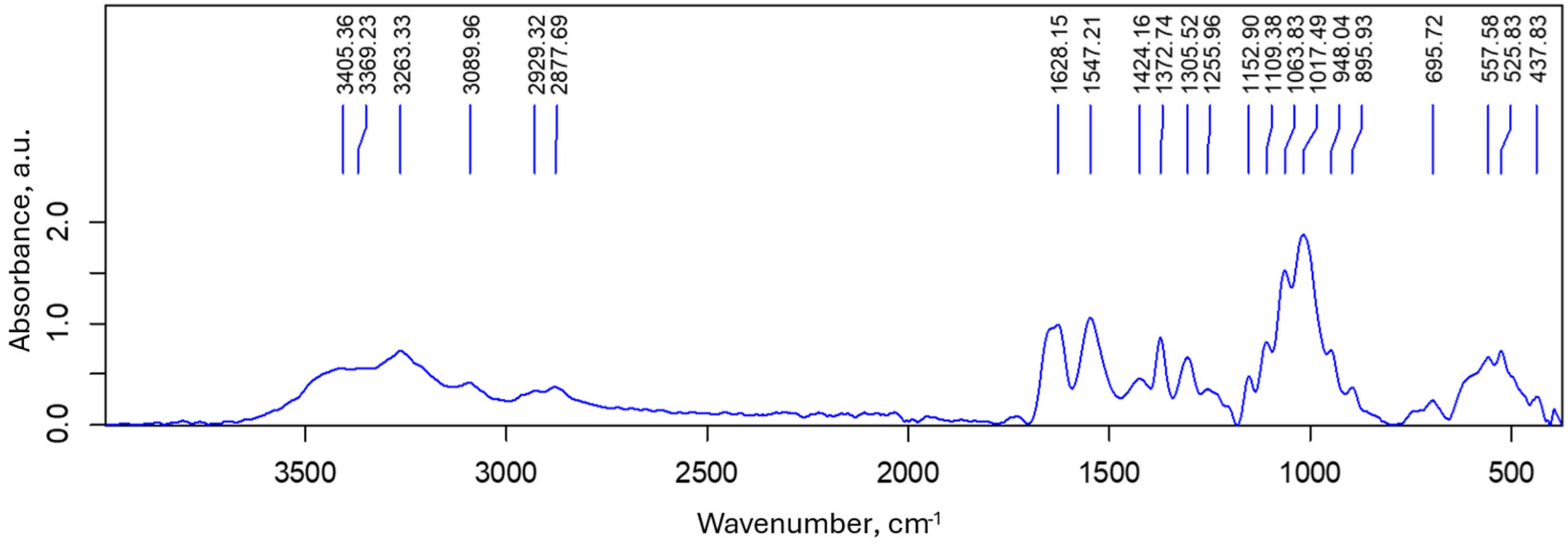

Figure S3. Fourier transform infrared (FTIR) spectrum of chitin, extracted with the help of ionic liquid. The broad band at 3405–3263 cm^{-1} corresponds to overlapping O–H and N–H stretching vibrations, indicative of extensive hydrogen bonding. Peaks at 3089–2878 cm^{-1} arise from C–H stretching vibrations. The characteristic amide I and amide II bands appear at 1628 and 1547 cm^{-1}, respectively, confirming the acetamide functionality of chitin. Bands at 1424–1375 cm^{-1} are assigned to CH_2 bending and C–H deformation modes. Strong absorptions in the 1153–895 cm^{-1} region correspond to C–O–C and C–O stretching vibrations of the polysaccharide backbone, while bands below 700 cm^{-1} are associated with skeletal vibrations of the chitin structure. The relative broadening of several bands is consistent with partially crystalline (amorphous-rich) chitin obtained via ionic-liquid-based processing.

2. Characterization of Chitin Nanomat Obtained by Electrospinning

2.1. ^{13}C CP-MAS SS NMR

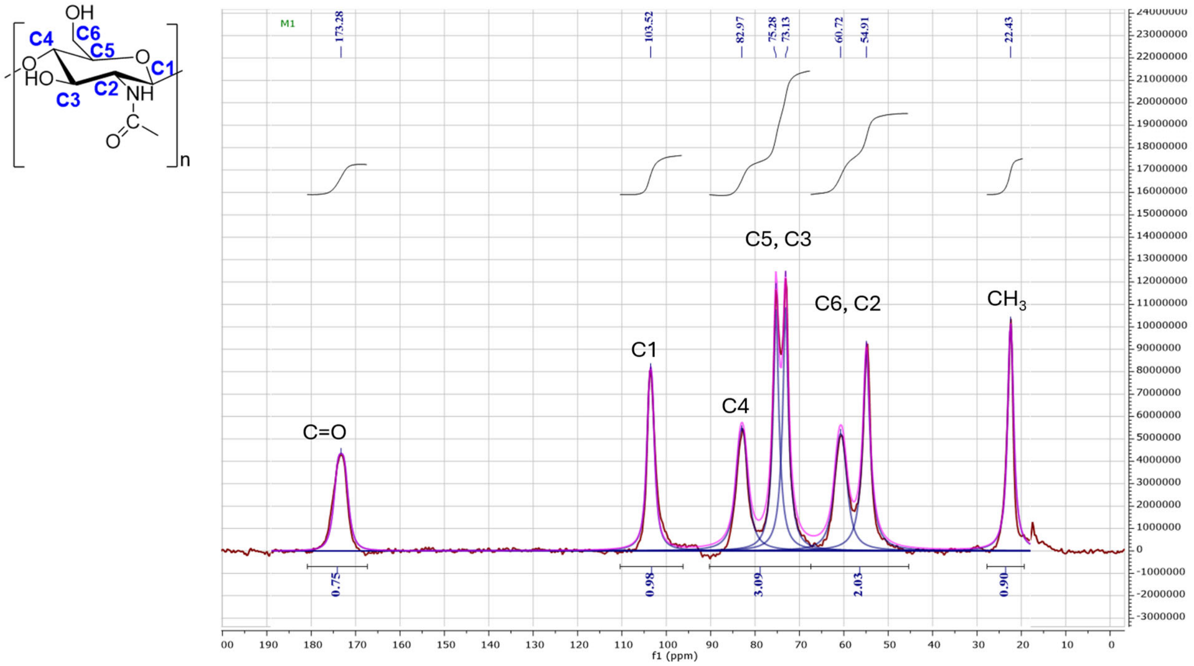

Figure S4. ^{13}C CP-MAS SS NMR of the prepared chitin nanomat. ^{13}C NMR peak list (δ, ppm → assignment): ~173.28 ppm → C=O (amide / acetyl carbonyl); ~103.52 ppm → C1 (anomeric carbon); ~82.97 ppm → C4; ~75.28 ppm → C5; ~73.13 ppm → C3; ~60.72 ppm → C6 (CH_2–OH); ~54.91 ppm → C2 (C–NHAc); ~22.43 ppm → CH_3 (acetyl methyl). Integrals (as reported in the spectrum): 0.75 (C=O), 0.98 (C1), 3.09 (C4+C5+C3), 2.03 (C6+C2), 0.90 (CH_3). (It should be noted that carbonyl signal intensities in ^{13}C CP/MAS are known to be non-quantitative; therefore, integral ratios involving the C=O resonance should be interpreted qualitatively.)

2.2. pXRD Diffractogram

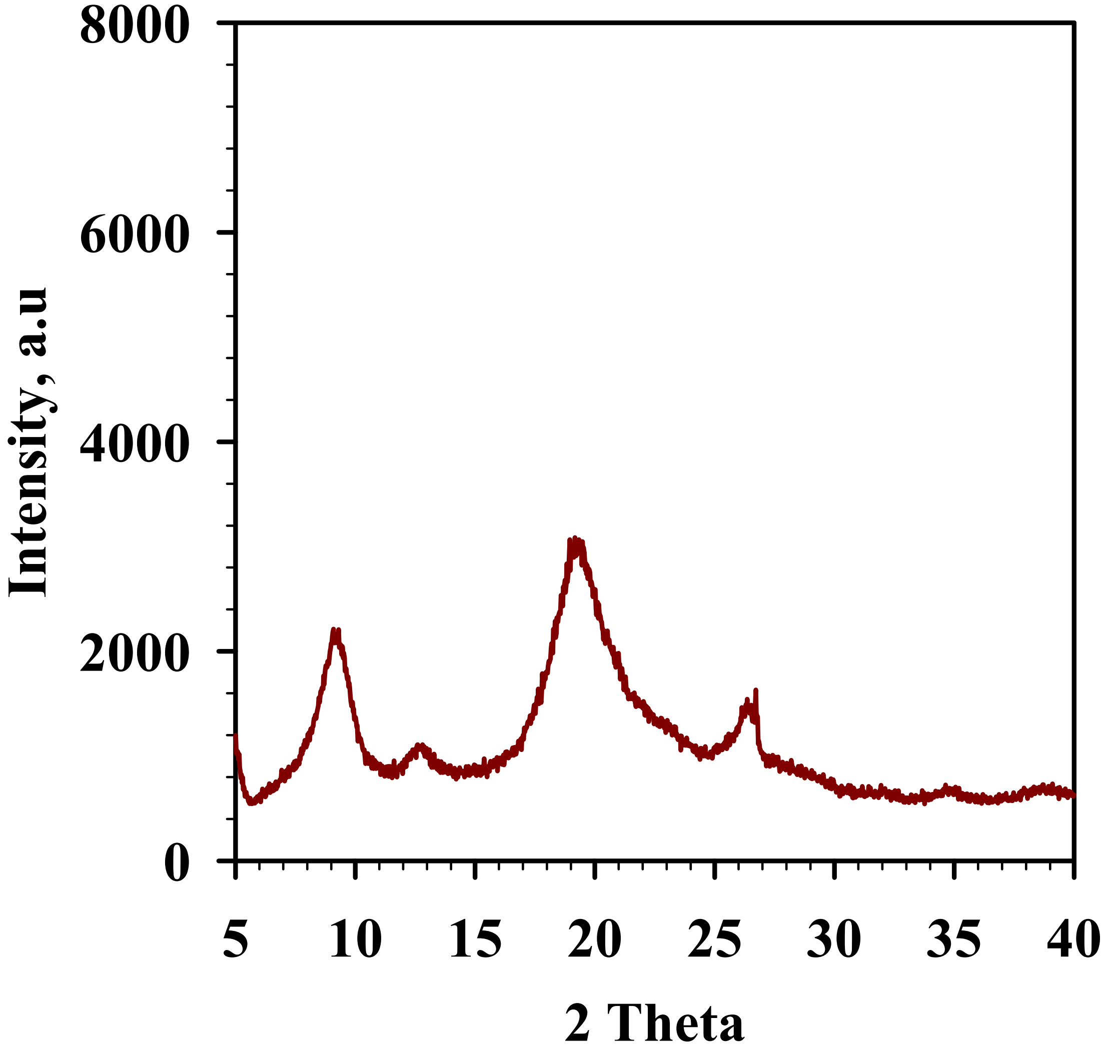

Figure S5. Powder X-ray diffraction (pXRD) pattern of the "as produced" chitin nanomat made of IL-extracted chitin–based material. Diffraction maxima are observed at **2θ = 9.15°, 12.50°, 19.07°, and 26.29°**, which are characteristic of the polymer with reduced crystallinity obtained via ionic-liquid processing relative to native chitin polymer. The broadened reflections and reduced long-range order are consistent with disruption of the native hydrogen-bonding network during IL dissolution and regeneration.

2.3.Fourier Transform Infra-Red Spectrum

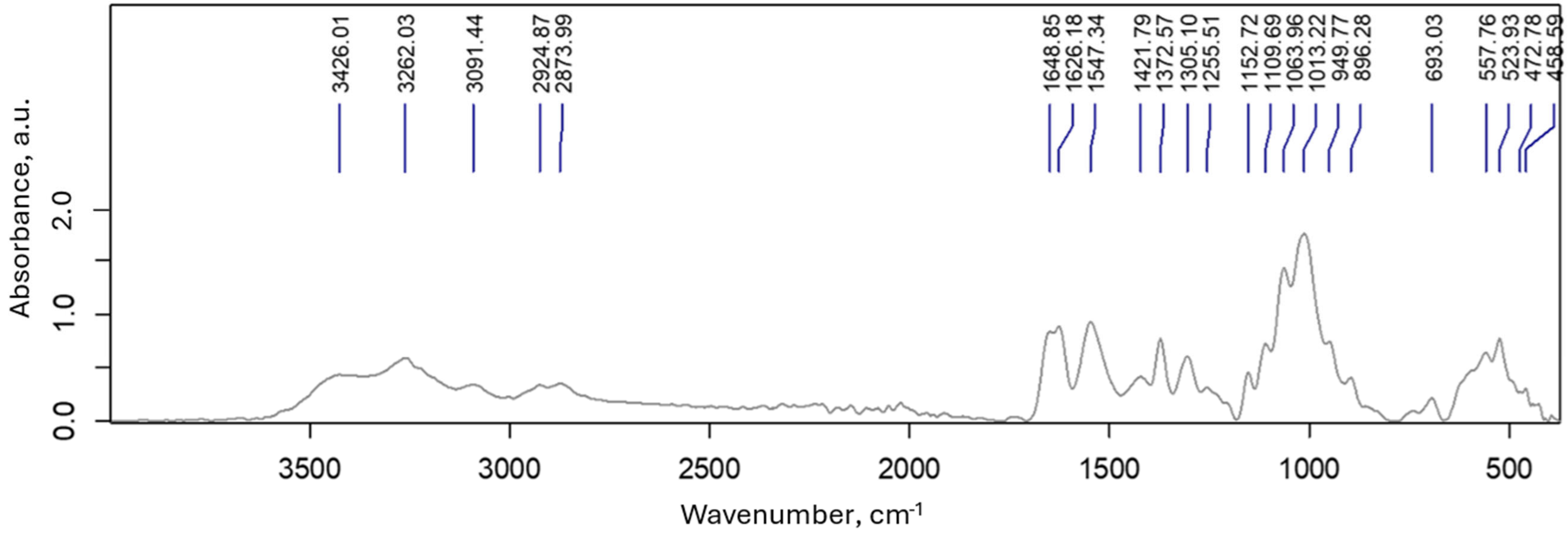

Figure S6. Fourier transform infrared (FTIR) spectrum of chitin, extracted with the help of ionic liquid. The broad band at 3405–3263 cm^{-1} corresponds to overlapping O–H and N–H stretching vibrations, indicative of extensive hydrogen bonding. Peaks at 3089–2878 cm^{-1} arise from C–H stretching vibrations. The characteristic amide I and amide II bands appear at 1628 and 1547 cm^{-1}, respectively, confirming the acetamide functionality of chitin. Bands at 1424–1375 cm^{-1} are assigned to CH_2 bending and C–H deformation modes. Strong absorptions in the 1153–895 cm^{-1} region correspond to C–O–C and C–O stretching vibrations of the polysaccharide backbone, while bands below 700 cm^{-1} are associated with skeletal vibrations of the chitin structure. The relative broadening of several bands is consistent with partially crystalline (amorphous-rich) chitin obtained via ionic-liquid-based processing.

2.4. Mercury Intrusion Porosimetry Data

Table S1. Mercury intrusion porosimetry parameters of representative electrospun chitin nanomats.

Sample	Sample mass (g)	Total intruded volume ($cm^3\ g^{-1}$)	MIP-derived surface area ($m^2\ g^{-1}$)	Porosity (%)	Volume-distribution mode pore diameter (μm)
1	0.0185	24.0785	5.0982	97.1968	17.49
2	0.0157	23.4604	5.3455	97.1250	18.61

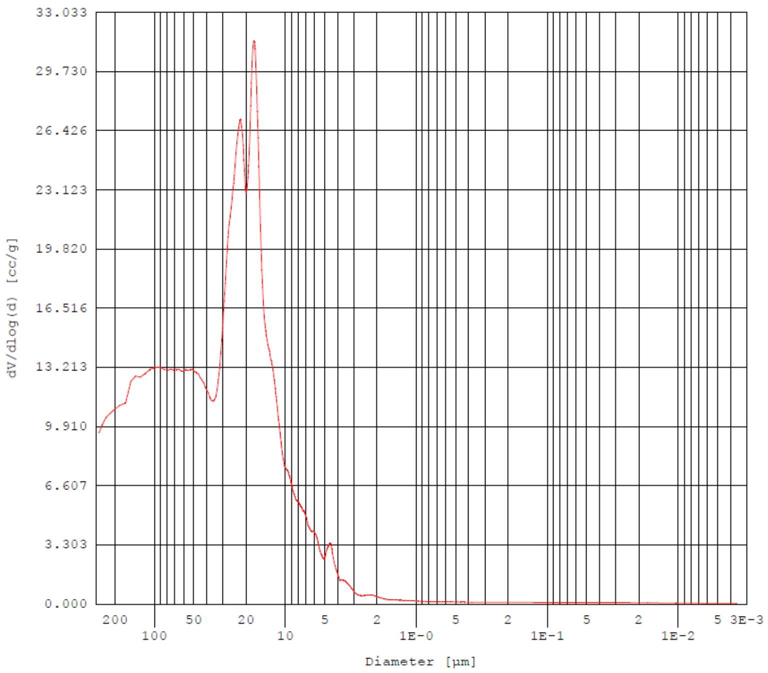

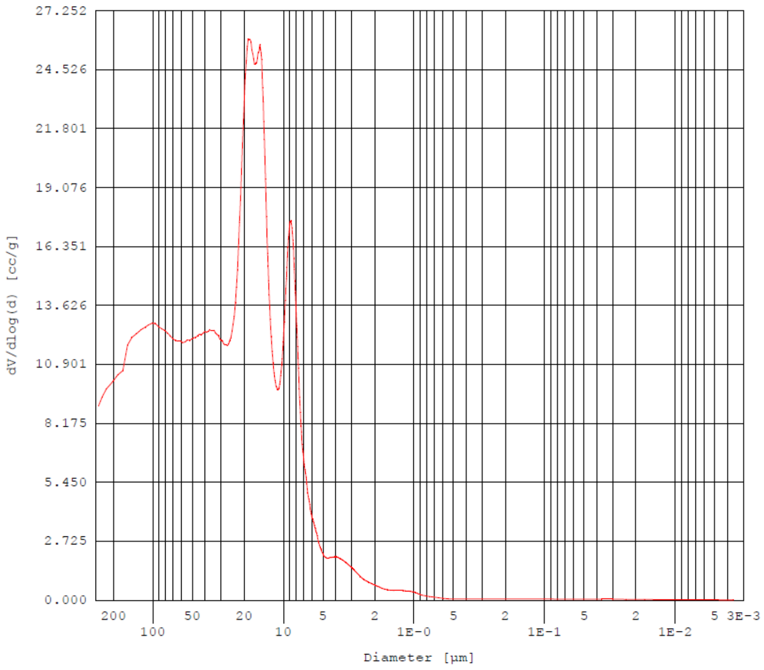

Figure S7. Mercury intrusion porosimetry pore-size distributions of representative electrospun chitin nanomats. Differential intrusion volume (−dV/dlogD) is plotted as a function of pore diameter. The principal volume-distribution modes occurred at approximately 17.49 and 18.61 μm for Samples 1 and 2, respectively.

3. Additional STEM Images of Metal Nanoparticles and Image-Based Dimensional Measurements

3.1. Palladium

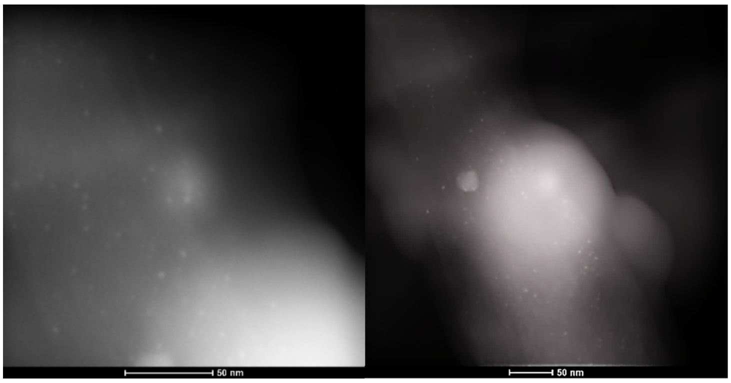

Figure S8. Additional STEM images of IL-processed chitin nanomats showing uniformly distributed Pd nanoparticles on nanofiber surfaces

Table S2. Image-Based Dimensional Measurements of Pd Nanoparticles (n = 20)

	Area	Mean	Min	Max	Angle	Length
1	0.802	115.699	108	120	-8.97	3.850
2	0.962	115.734	111	121	74.75	4.608
3	1.323	120.616	114	127	0.00	6.329
4	0.802	124.112	120	127	-90.00	3.797
5	0.682	107.844	104	110	10.62	3.227
6	0.561	101.070	97	104	0.00	2.532
7	0.802	96.998	93	100	0.00	3.797
8	0.561	104.106	100	107	-41.99	2.685
9	0.842	104.145	101	107	39.09	4.053
10	0.481	97.329	96	99	56.31	2.282
11	0.330	154.697	135	169	0.00	3.201
12	0.310	131.620	119	144	-23.20	3.046
13	0.230	163.731	155	171	-21.80	2.154
14	0.250	168.640	159	177	-90.00	2.400
15	0.280	138.631	122	152	26.57	2.683
16	0.170	156.250	146	164	-14.04	1.649
17	0.290	122.586	101	135	0.00	2.800
18	0.380	147.256	136	159	-49.40	3.688
19	0.250	129.560	117	140	90.00	2.400
20	0.330	107.182	93	116	0.00	3.200
					**Average**	**3.219**
					**STDev**	**1.02**

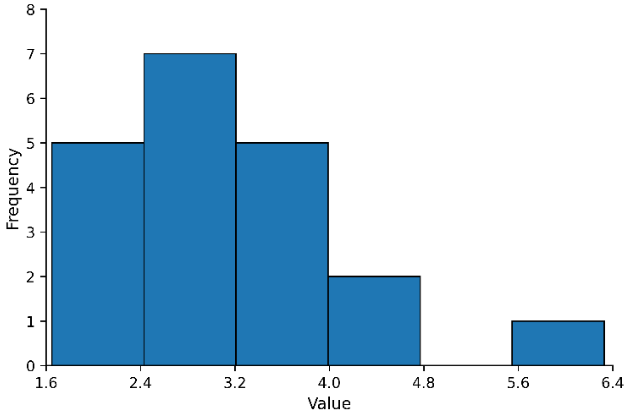

4.

Figure S9. Distribution of image-based diameter measurements for Pd nanoparticles (n = 20). Histogram bin width = 0.8 nm.

3.2. Gold

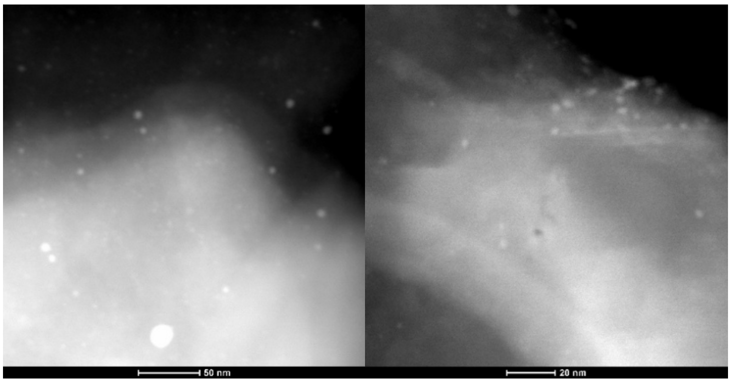

Figure S10. Additional STEM images of IL-processed chitin nanomats showing uniformly distributed Au nanoparticles on nanofiber surfaces

Table S3. Image-Based Dimensional Measurements of Au Nanoparticles (n = 25)

	Area	Mean	Min	Max	Angle	Length
1	1.605	253.512	235	254	-25.20	10.923
2	1.732	247.829	195	254	-8.53	11.757
3	0.993	231.631	158	254	-15.26	6.628
4	0.591	219.116	186	233	26.57	3.900
5	0.887	248.322	222	254	-11.31	5.929
6	1.732	230.921	95	254	-8.537	11.757
7	0.697	215.030	188	235	0.00	4.651
8	0.570	248.933	225	254	-38.66	3.722
9	1.120	234.075	148	254	0.00	7.558
10	1.500	246.678	171	254	23.63	10.153
11	1.605	252.415	214	254	105.52	10.861
12	0.845	210.183	164	230	-23.96	5.726
13	0.655	229.546	197	249	-23.20	4.427
14	0.697	247.879	231	254	0.00	4.651

15	0.739	227.741	188	244	-20.56	4.967
16	1.011	126.431	110	138	-24.44	6.944
17	0.599	247.690	229	254	-8.13	4.064
18	0.970	113.070	84	134	-37.88	6.552
19	0.784	82.843	66	95	40.60	5.298
20	0.578	105.802	94	114	-26.57	3.855
21	0.619	248.120	231	254	33.69	4.144
22	0.867	251.931	236	254	-11.31	5.861
23	0.516	170.200	163	176	0.00	3.448
24	0.826	123.316	116	129	-66.04	5.660
					**Average**	**6.393**
					**STDev**	**2.64**

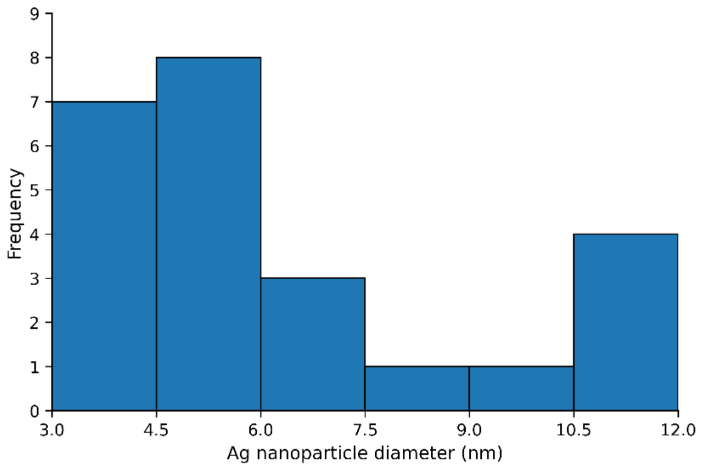

Figure S11. Distribution of image-based diameter measurements for Au nanoparticles (n = 24). Histogram bin width = 1.5 nm.

3.3. Silver

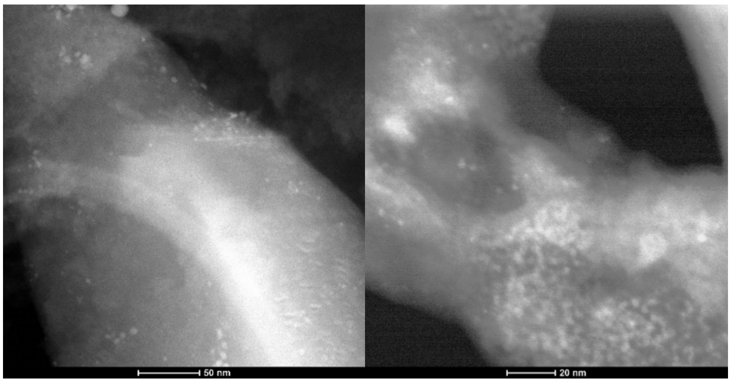

Figure S12. Additional STEM images of IL-processed chitin nanomats showing uniformly distributed Ag nanoparticles on nanofiber surfaces

Table S4. Image-Based Dimensional Measurements of Ag Nanoparticles (n = 30)

	**Area**	**Mean**	**Min**	**Max**	**Angle**	**Length**
1	0.846	135.397	109	145	-36.87	5.747
2	0.846	135.397	109	145	-36.87	5.747
3	0.681	123.402	99	135	-29.75	4.633
4	0.392	109.952	86	124	45.00	2.569
5	0.289	80.221	73	83	0.00	1.816
6	0.289	82.102	74	90	0.00	1.816
7	0.289	50.310	41	57	0.00	1.816
8	0.206	103.274	91	109	0.00	1.362
9	0.537	214.783	193	228	6.843	3.661
10	0.475	105.248	86	121	-7.431	3.211
11	0.413	102.590	91	108	0.00	2.725
12	0.206	168.878	166	171	0.00	1.362
13	0.351	161.390	150	172	0.00	2.270
14	0.495	145.813	130	160	34.38	3.275
15	0.681	140.592	112	158	0.00	4.541
16	0.413	147.803	136	161	8.973	2.762
17	0.413	136.352	117	150	11.889	2.762
18	0.206	109.011	101	115	0.00	1.362
19	0.114	195.219	173	228	11.31	1.500
20	0.135	232.720	181	254	0.00	1.765
21	0.146	155.387	133	174	38.66	1.883
22	0.124	226.346	206	242	68.20	1.584
23	0.178	137.002	116	166	60.26	2.371
24	0.206	118.976	96	156	-6.34	2.703
25	0.134	189.245	172	217	30.96	1.741
26	0.145	250.406	235	254	-18.44	1.888
27	0.173	156.071	128	182	-23.20	2.273
28	0.195	215.009	190	240	-20.56	2.550
29	0.117	202.640	167	225	53.13	1.493
30	0.162	88.167	60	114	8.13	2.111
					**Average**	**2.577**
					**STDev**	**1.19**

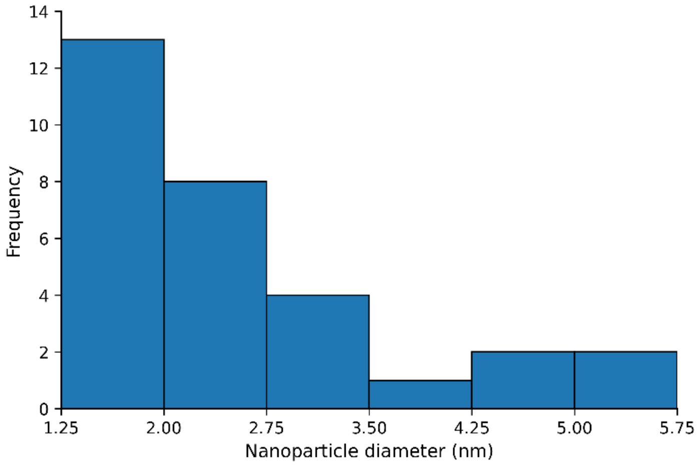

Figure S13. Distribution of image-based diameter measurements for Ag nanoparticles (n = 30). Histogram bin width = 0.75 nm.

3.4.Palladium-Gold

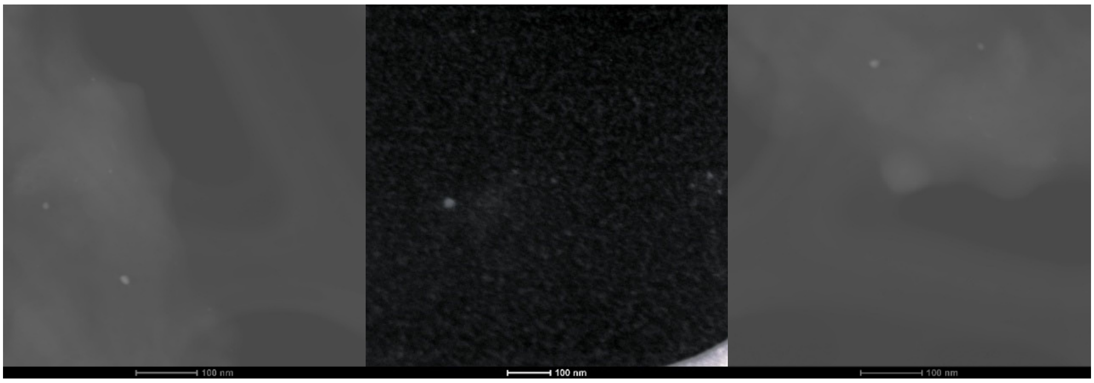

Figure S14. Additional STEM images of IL-processed chitin nanomats showing uniformly distributed Pd-Au nanoparticles on nanofiber surfaces

4. Energy-Dispersive X-ray Spectroscopy (EDS)

4.1. Palladium

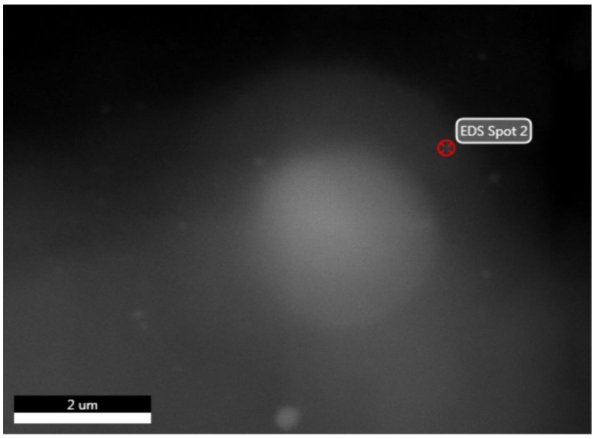

kV: 200.1
Mag: 20000
Takeoff: 20.4
Live Time (s): 30
Amp Time (µs): 3.84
Resolution (eV): 128.2

EDS Spot 2

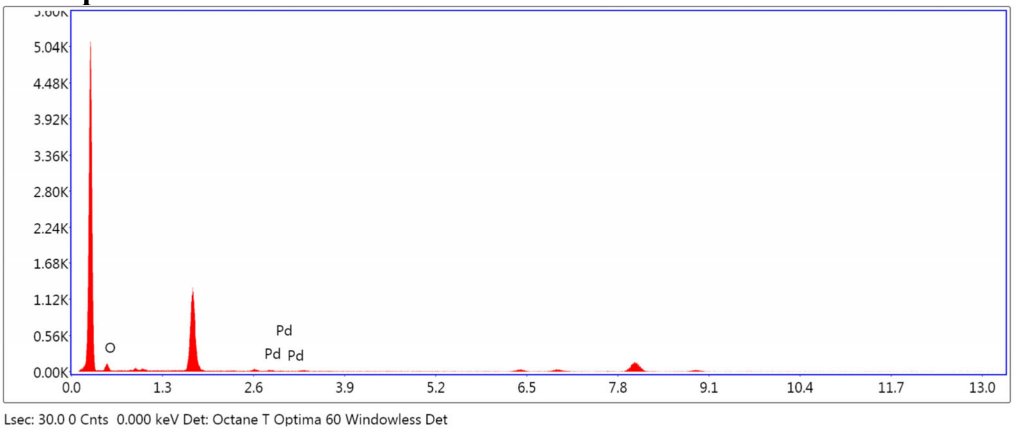

Element	Weight %	Atomic %	Net Int.	Net Error %	KABFactor
O K	17.12	57.87	8.50	10.14	1
PdL	82.88	42.13	7.57	8.67	5.44

Figure S15. EDS Spectra of Pd–Chitin Nanomats. Energy-dispersive X-ray spectroscopy (EDS) analysis collected at 200 kV confirms the presence of Pd in the nanomats. Quantitative EDS results (verify consistent Pd content and negligible adventitious elements.

4.2.Gold

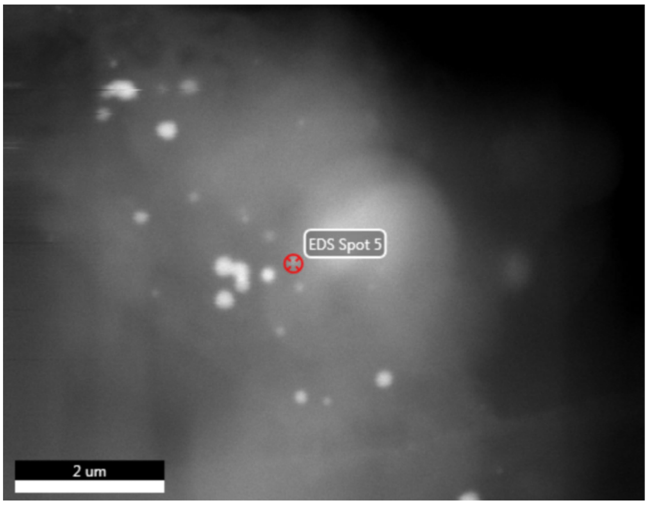

kV:	200.1
Mag:	20000
Takeoff:	20.4
Live Time (s):	30
Amp Time (µs):	0.96
Resolution (eV):	135.6

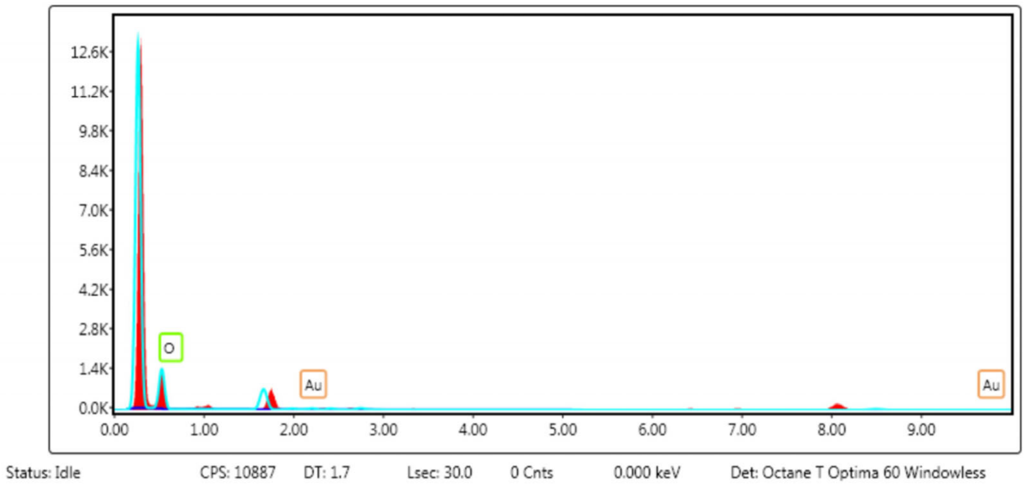

Element	Weight %	Atomic %	KAB Factor	Net Int.	Net Error %
O K	94.73	99.55	1	472.33	0.89
Au L	5.27	0.45	10.24	2.57	24.26

Figure S16. EDS Spectra of Au–Chitin Nanomats. Energy-dispersive X-ray spectroscopy (EDS) analysis collected at 200 kV confirms the presence of Au in the nanomats.

5. TEM Data for the Au–Pd–Chitin Nanomat

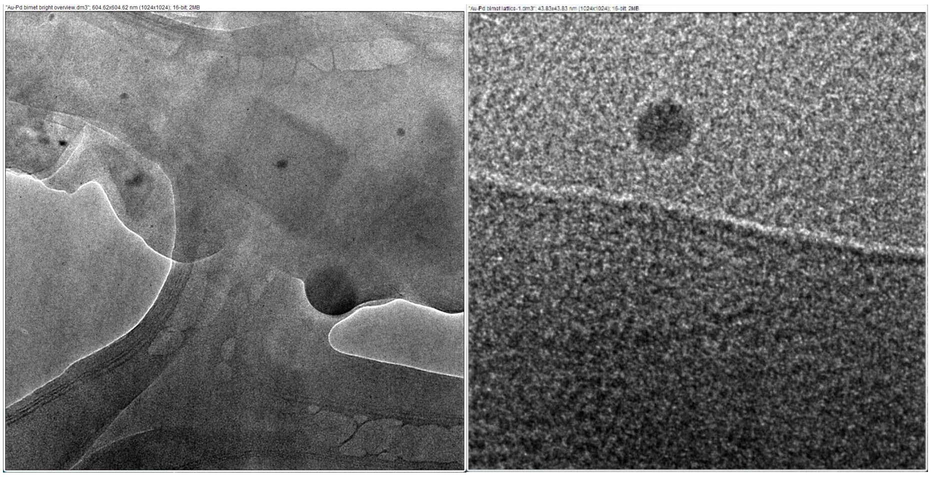

Figure S17. Left: Low-magnification TEM overview of the Au–Pd–chitin nanomats showing the ultrathin chitin fiber network and the uniform distribution of electron-dense bimetallic nanoparticles across the film. Right: High-resolution TEM micrograph of an individual Au–Pd nanoparticle anchored on the chitin surface, highlighting its well-defined spherical morphology and crystalline contrast. This region was used for lattice-resolved FFT analysis of the Au–Pd alloy structure.

6. Gas Chromatography–Mass Spectrometry (GC–MS) Results

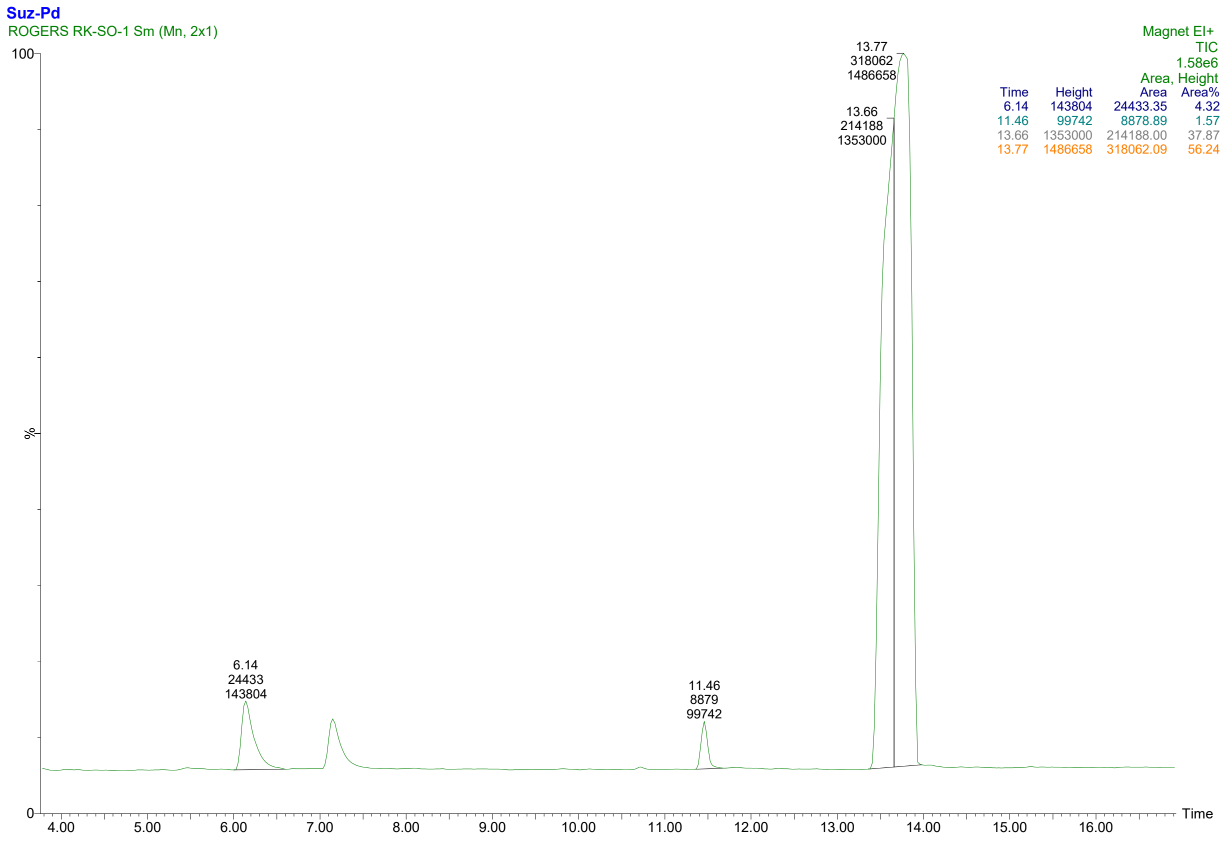

Figure S18. GC-MS after Suzuki Reaction: Pd NP-Chitin

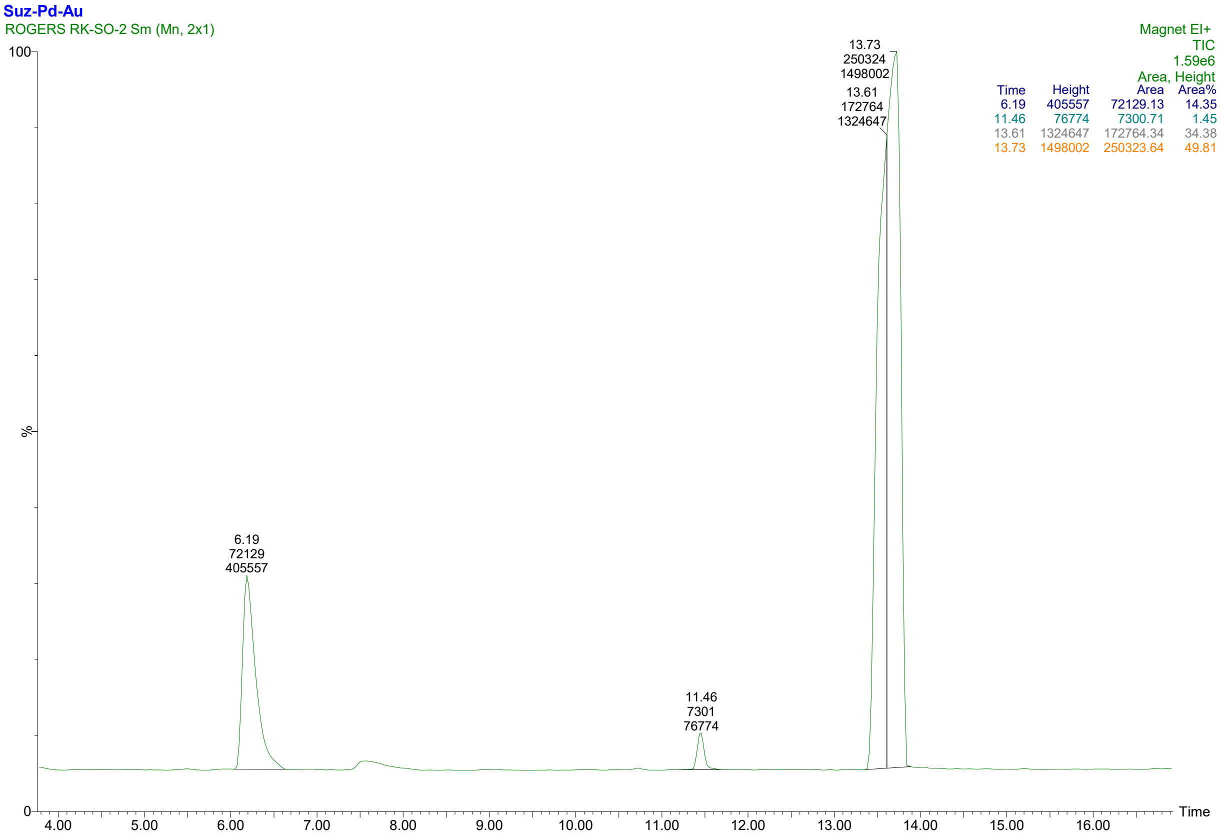

Figure S19. GC-MS after Suzuki Reaction: Pd-Au NP-Chitin.
